# Ring-opening reaction of 2,5-dioctyldithieno[2,3-*b*:3',2'-*d*]thiophene in the presence of aryllithium reagents

**DOI:** 10.3762/bjoc.9.87

**Published:** 2013-04-19

**Authors:** Hao Zhong, Jianwu Shi, Jianxun Kang, Shaomin Wang, Xinming Liu, Hua Wang

**Affiliations:** 1 Key Lab for Special Functional Materials of Ministry of Education, Henan University, Kaifeng, 475004, China; 2 Department of Chemistry, Zhengzhou University, Zhengzhou, 450001, China

**Keywords:** aryllithium reagents, 2'-arylthio-3,3'-bithiophene-2-carbaldehyde, dithieno[2,3-*b*:3',2'-*d*]thiophene, nucleophilicity, ring-opening reaction

## Abstract

In this paper, the ring-opening reaction of 2,5-dioctyldithieno[2,3-*b*:3',2'-*d*]thiophene with aryllithium in THF at low temperature to generate 2'-arylthio-3,3'-bithiophene-2-carbaldehydes is studied. Nine examples are explored and all the products are characterized by ^1^H NMR, ^13^C NMR and HRMS. The relative relationship between the structures of aryl groups and the efficiency of ring-opening reactions are discussed.

## Introduction

**Scheme 1:**
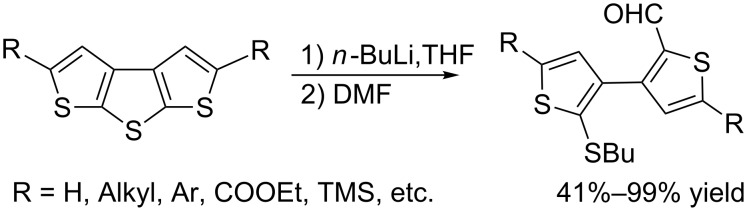
The ring-opening reaction of symmetric 2,5-disubstituted-dithieno[2,3-*b*:3',2'-*d*]thiophenes in the presence of *n*-BuLi in THF.

Due to the promising optical and electrical properties, the derivatives of dithieno[2,3-*b*:3',2'-*d*]thiophene (DTT), as one type of fused oligothiophene, have shown their potential applications in organic electronics [1–4]. The work on the synthesis of DTT derivatives and the chemical stability of the DTT core is of particular interest. To construct DTT functional materials, deprotonation of DTT with organolithium reagents seems to be one of the most important approaches. However, the ring-opening reaction of DTT leading to the cleavage of the center ring can be observed in the presence of *n*-BuLi. In our previous work, we reported the synthesis of a series of symmetric substituted dithieno[2,3-*b*:3',2'-*d*]thiophenes and their ring-opening reactions in the presence of *n*-BuLi. The 3,3'-bithiophene-2-carbaldehydes were generated after quenching with an electrophile, i.e., dry DMF (Scheme 1) [5].

The uncommon ring opening of fused thiophene derivatives in the presence of *n*-BuLi, though it has been reported, does not draw as much attention as the ring opening of other heterocyclic compounds [6–9]. The limited number of reports include derivatives of benzo[*b*]thiophene [10–12], thieno[3,2-*d*]thiazole [10–11], and thieno[3,2-*b*]thiophene [13]. More recently, Nenajdenko et al. reported that fused thieno[2,3-*b*]thiophenes and some [3,2-*b*]-fused oligothiophenes were attacked by organo-lithium reagents resulting in the cleavage of thiophene rings [14]. They found when competitive deprotonation of the substrate was possible, high selectivity towards the ring opening was observed with *n*-BuLi when compared with other organolithium reagents. However, most of these ring-opening reactions mentioned above take place by using *n*-BuLi as the nucleophile to attack the sulfur atoms of thiophenes. Other organolithium reagents have rarely been employed for this kind of reaction. Furthermore, the relationship between the nucleophilicity of organolithium reagents and the efficiency of the ring opening of fused thiophenes has not been discussed.

In this paper, we present the ring opening of 2,5-dioctyldithieno[2,3-*b*:3',2'-*d*]thiophene (**1**) with nine aryllithium reagents and the characterization of the nine corresponding ring-opening products. These studies should facilitate the understanding of the chemical stability of dithieno[2,3-*b*:3',2'-*d*]thiophene, which may be of importance in both organic chemistry and materials science. Furthermore, we show a novel method for the synthesis of 2'-arylthio-3,3'-bithiophene-2-carbaldehydes based on the ring-opening reaction.

## Results and Discussion

### Ring-opening reaction of 2,5-dioctyldithieno[2,3-*b*:3',2'-*d*]thiophene in the presence of aryllithium reagents

The aryllithium reagents (Ar–Li) were obtained from the metal–halogen exchange of Ar–Br and *n*-BuLi. To avoid the possible influence from excess *n*-BuLi, 1.4 equiv Ar–Br was treated with 1.3 equiv *n*-BuLi at −78 °C for 2 h to make sure that only excess Ar–Li (1.3 equiv) was employed for the ring-opening reaction. Then a solution of **1** was added at −78 °C, at which point the reaction mixture was slowly warmed to −30 °C for 3 h. After quenching with dry DMF, the corresponding ring-opening products 2'-arylthio-3,3'-bithiophene-2-carbaldehydes were obtained.

Nine aryllithium reagents were employed for the ring-opening reaction of **1** as shown in Table 1. The molecular structures and the effects of electron-donating groups (EDG) and electron-withdrawing groups (EWG) of the aryllithium reagents were studied. Compared to the compound **2d** (Table 1, entry 4) bearing EWG groups, the compounds **2b**, **2c** and **2e** (Table 1, entries 2, 3 and 5), all of which have EDG groups, generated higher yields of products, namely 73% (**3b**), 74% (**3c**) and 83% (**3e**), respectively. In the case of **2b**, not only was **3b** obtained, but also a byproduct with similar polarity, 2'-butylsulfanyl-5,5'-dioctyl-[3,3'-bithiophene]-2-carbaldehyde [5], was generated when the metal–halogen exchange temperature was set to −78 °C. If the reaction temperature of the metal–halogen exchange was set to −78 °C first, and then warmed up to −30 °C for 3 h, only **3b** could be generated in 70% yield (Table 1, entry 3). The byproduct formed in the case of **2b** implies that the metal–halogen exchange cannot be completed at −78 °C.

**Table 1:** The ring opening of 2,5-dioctyldithieno[2,3-*b*:3’,2’-*d*]thiophene (**1**) with aryllithium reagents **2a**–**i**.

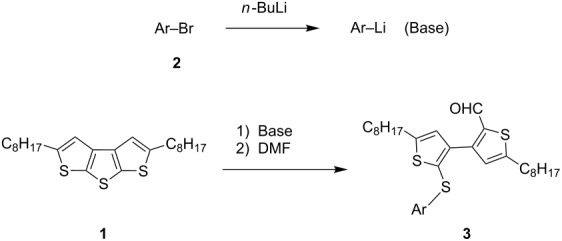			

Entry	Ar–Br	Yield (%)^a^	Product

1	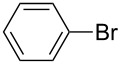 **2a**	88	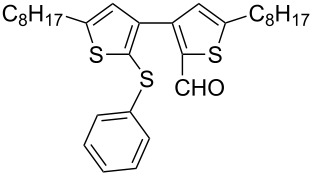 **3a**
2	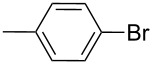 **2b**	70^b^	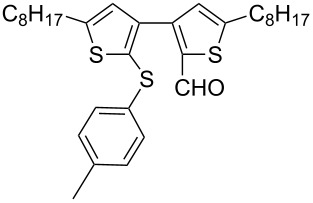 **3b**
3	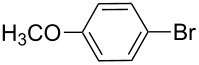 **2c**	71^b^	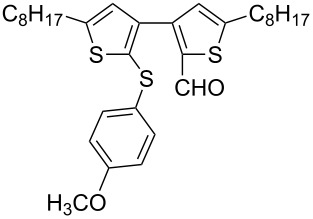 **3c**
4	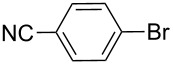 **2d**	42	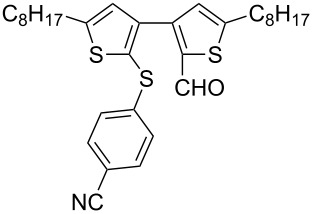 **3d**
5	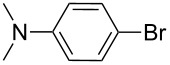 **2e**	83	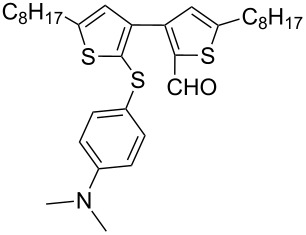 **3e**
6	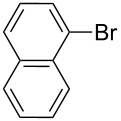 **2f**	61	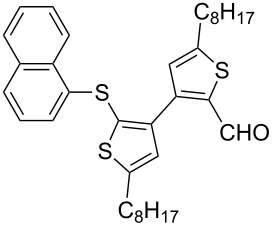 **3f**
7	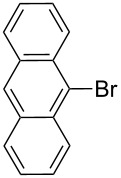 **2g**	56	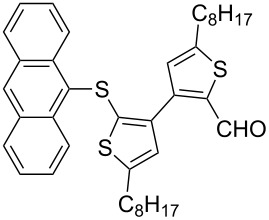 **3g**
8	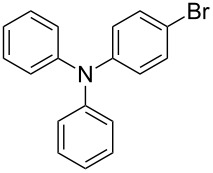 **2h**	76	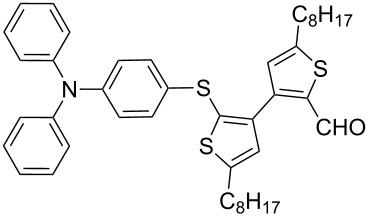 **3h**
9	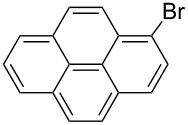 **2i**	48^c^	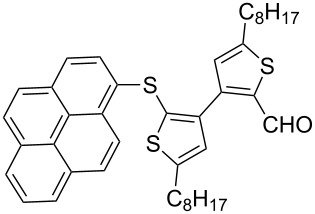 **3i**

^a^Yield of the isolated product. ^b^*n*-BuLi was added at −78 °C then warmed up to −30 °C for 3 h. ^c^*t*-BuLi was employed instead of *n*-BuLi.

Similar to the case of **2e** bearing the EDG group of triphenylamine, **2h** also gave a good yield of **3h** (76%, Table 1, entry 8). These results indicate that an increase of the electron donating character of the substituted aryl groups leads to a higher nucleophilicity of the aryllithium reagents and thereby promotes the process of a ring-opening reaction. On the other hand, in the case of **2d**, which has a cyano group, a poor yield of **3d** (42%, Table 1, entry 4) was obtained due to the high stability of cyano-aryl anion with a low nucleophilicity.

The steric hinderance effect can be observed in the case of **2a**, **2f** and **2g** (Table 1, entries 1, 6 and 7). When the bulkiness of aryl groups increased from benzene to anthracene, the yields of ring-opening products decreased from **3a** (88%), to **3f** (61%) and to **3g** (56%). Therefore, the aryllithium reagents with bulky groups, such as anthryllithium, generate lower yields of ring-opening products than phenyllithium as the nucleophilic reagent.

**Scheme 2:**
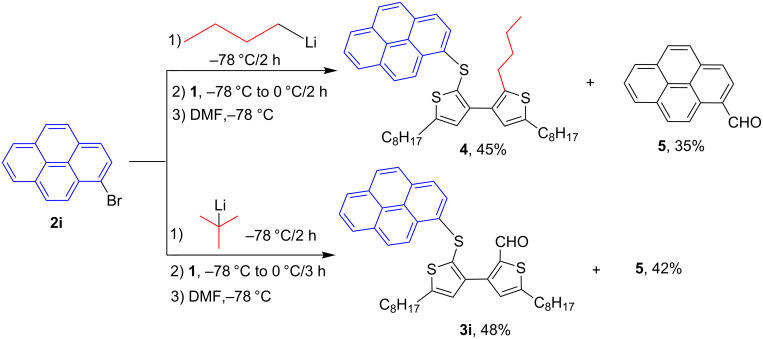
The ring-opening reaction of **1** in the presence of *n*-BuLi and *t*-BuLi employed for metal–halogen exchange.

**Figure 1:**
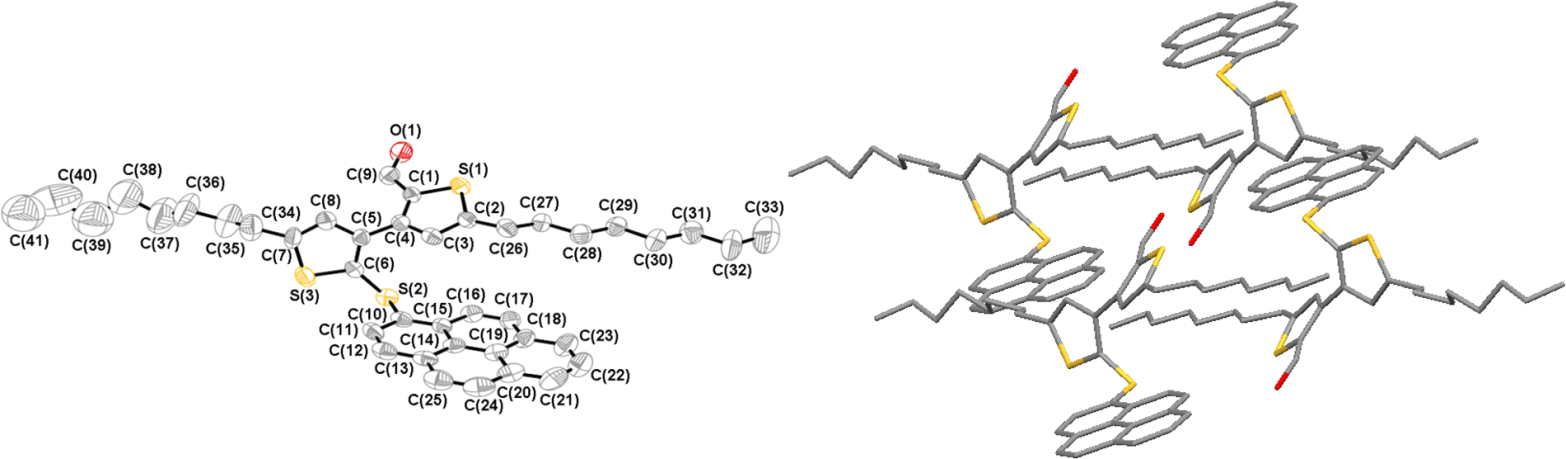
Crystallographic structure of **3i** (left, top view) and crystal packing (right). Carbon, silicon, oxygen and sulfur atoms are depicted with thermal ellipsoids set at 50% probability level, and all hydrogen atoms are omitted for clarity.

An interesting result was observed for the metal–halogen exchange of 1-bromopyrene (**2i**) with *n*-BuLi or *t*-BuLi at −78 °C, which delivered different types of ring-opening products (Scheme 2). When *n*-BuLi was used for the metal–halogen exchange, the reaction temperature was set at 0 °C for the ring-opening reaction of **1** for 3 h. Instead of the expected product, however, an unexpected ring-opening product 2'-butyl-5,5'-dioctyl-2-(1-pyrenylthio)-3,3'-bithiophene (**4**, 45%) along with 1-pyrenecarboxaldehyde (**5**, 35%) was generated when the reaction mixture was quenched with dry DMF. If *t*-BuLi was employed for the metal–halogen exchange, only anticipated product **3i** was obtained in 48% yield and no **4** was observed. The structure of **3i** was confirmed by a single-crystal structure analysis (Figure 1).

In our work, the metal–halogen exchange of 1-bromopyrene (**2i**) with *n*-BuLi or *t*-BuLi at −78 °C delivered different types of ring-opening products. We believe that the two metal–halogen exchange processes went to complete conversion. The formation of **4** is possibly due to the in situ generation of *n*-BuBr that is trapped by the carbanion generated from an attack of pyrenyllithium (PyLi) on the central sulfur atom of **1**. The presence of the pyrene carbanion intermediate is responsible for the formation of **5** by dry DMF quenching. However, if *t*-BuLi was used for the metal–halogen exchange, *t*-BuBr could not be efficiently generated due to the fast elimination occurring between the formed *t*-BuBr and another equivalent of *t*-BuLi. The formed pyrene–Li attacks the sulfur center of **1** and generates the carbanion intermediate via a ring-opening mechanism, which was quenched only by dry DMF, resulting in **3i**.

### Crystal structure of 2'-(1-pyrenylsulfanyl)-5,5’-dioctyl-[3,3’-bithiophene]-2-carbaldehyde (**3i**)

The crystallographic structure of **3i** is shown in Figure 1 [15]. The crystal of **3i** belongs to the triclinic space group P−1. The two thiophene rings are linked together in the molecule with a torsional angle (C3–C4–C5–C6) of 55.4° and a dihedral angle of 56.1°. The pyrenylsulfanyl group is not coplanar to the neighboring thiophene ring with a dihedral angle of 79.5° and a torsion angle (C5–C6–S2–C10) of 89.7°. There are short contacts of hydrogen bonding found in the crystal packing. However, no π–π interaction was observed between two pyrene rings, as usually seen in the crystal (Figure 1, right).

## Conclusion

In summary, aryllithium reagents are suitable for the ring opening of 2,5-dioctyldithieno[2,3-*b*:3',2'-*d*]thiophene. We obtained nine ring-opening products. Their yields indicated that strong nucleophilicity of the aryllithium reagents can intensify the efficiency of the ring-opening process, and the steric effect is another factor that could influence the yield. This ring opening can be applied extensively to derivatives including 2'-arylthio-3,3'-dithiophenyl-2-aldehydes, which may provide access to a broad range of compounds for pharmaceutical chemistry, organic chemistry and materials science.

## Experimental

**Synthesis of 2'-phenylsulfanyl-5,5'-dioctyl-[3,3'-bithiophene]-2-carbaldehyde (3a):** To a solution of **2a** (49.7 mg, 0.32 mmol, 1.4 equiv) in dry THF (10 mL), *n*-BuLi (2.38 M in hexane, 0.12 mL, 0.29 mmol, 1.3 equiv) was added dropwise at −78 °C. After stirring at −78 °C for 2 h, a solution of **1** (95.0 mg, 0.23 mmol, 1.0 equiv) in dry THF (10 mL) was added dropwise. The mixture was slowly warmed to −30 °C for 3 h, then dry DMF (0.04 mL, 0.45 mmol, 2.0 equiv) was added dropwise at −78 °C, and the reaction mixture was slowly warmed to ambient temperature overnight. After quenching with H_2_O (30 mL), the reaction mixture was extracted with CHCl_3_ (2 × 30 mL) and then washed with H_2_O (30 mL). After drying over MgSO_4_, the solvent was removed in vacuum. The residue was purified by column chromatography on silica gel with petrol ether (60–90 °C)/chloroform (1:4, v/v) as eluents to yield **3a** (105.1 mg, 88.4%) as a light yellow oil. ^1^H NMR (400 MHz, CDCl_3_) δ 9.70 (s, 1H), 7.22–7.19 (m, 2H), 7.14–7.07 (m, 3H), 6.87 (s, 1H), 6.82 (s, 1H), 2.82 (t, *J* = 7.6 Hz, 2H), 2.79 (d, *J* = 7.4 Hz, 2H), 1.74–1.61 (m, 4H), 1.40–1.27 (m, 20H), 0.90–0.87 (m, 6H); ^13^C NMR (100 MHz, CDCl_3_) δ 183.03, 155.28, 150.54, 144.49, 139.86, 137.93, 136.87, 128.83, 128.30, 127.74, 127.16, 126.06, 126.02, 31.71, 31.12, 30.85, 30.56, 30.31, 29.14, 29.11, 29.06, 29.04, 28.98, 28.82, 22.54, 14.01; IR (KBr): 2955, 2926, 2855 (C-H), 1661 (C=O) cm^−1^; HRMS–EI *m*/*z*: [M^+^ + Na] calcd for C_31_H_42_OS_3_Na, 549.2293; found, 549.2290.

**Synthesis of 2'-(4-methylphenylsulfanyl)-5,5'-dioctyl-[3,3'-bithiophene]-2-carbaldehyde (3b):** The same procedure was used as for the synthesis of **3a** except that after the addition of *n*-BuLi, the reaction temperature was raised to −30 °C for 3 h, and then cooled back to −78 °C before the addition of **1**. From the reaction on the 54.2 mg scale of **2b**, 90.0 mg (70.3%) of **3b** was obtained as a yellow oil. ^1^H NMR (400 MHz, CDCl_3_) δ 9.69 (s, 1H), 7.02 (s , 4H), 6.84 (d, *J* = 1.3 Hz, 2H), 2.80 (t, *J* = 7.2 Hz, 2H), 2.79 (t, *J* = 7.2 Hz, 2H), 2.28 (s, 3H), 1.73–1.63 (m, 4H), 1.39–1.28 (m, 20H), 0.88 (t, *J* = 6.8 Hz, 6H); ^13^C NMR (100 MHz, CDCl_3_) δ 183.28, 155.38, 150.07, 144.78, 139.14, 136.86, 136.41, 134.09, 129.70, 128.44, 128.13, 127.65, 127.42, 31.77, 31.18, 30.96, 30.66, 30.35, 29.20, 29.19, 29.12, 29.04, 28.92, 22.60, 20.92, 14.07; IR (KBr): 2956, 2926, 2855 (C-H), 1661 (C=O) cm^−1^; HRMS–EI *m*/*z*: [M^+^ + Na] calcd for C_32_H_44_OS_3_Na, 563.2430; found, 563.2437.

**Synthesis of 2'-(4-methoxylphenylsulfanyl)-5,5'-dioctyl-[3,3'-bithiophene]-2-carbaldehyde (3c):** The same procedure was used as for the synthesis of **3b**. From the reaction on the 61.9 mg scale of **2c**, 92.9 mg (70.5%) of **3c** was obtained as a yellow oil. ^1^H NMR (400 MHz, CDCl_3_) δ 9.68 (s, 1H), 7.13–7.10 (m, 2H), 6.85 (s, 1H), 6.78 (s, 1H), 6.77–6.74 (m, 2H), 3.75 (s, 3H), 2.82 (t, *J* = 7.6 Hz, 2H), 2.76 (t, *J* = 7.7 Hz, 2H), 1.73–1.63 (m, 4H), 1.37–1.27 (m, 20H), 0.90–0.86 (m, 6H); ^13^C NMR (100 MHz, CDCl_3_) δ 183.34, 158.98, 155.41, 149.24, 144.91, 137.82, 136.78, 131.34, 129.42, 128.46, 127.50, 127.47, 114.57, 55.21, 31.76, 31.17, 31,02, 30.68, 30,29, 29.19, 29.11, 29.03, 28.94, 22.59, 14.06; IR (KBr): 2958, 2926, 2856 (C-H), 1661 (C=O) cm^−1^; HRMS–EI *m*/*z*: [M^+^ + Na] calcd for C_32_H_44_O_2_S_3_Na, 579.2395; found, 579.2396.

**Synthesis of 2'-(4-cyanolphenylsulfanyl)-5,5'-dioctyl-[3,3'-bithiophene]-2-carbaldehyde (3d):** The same procedure was used as for the synthesis of **3a**. From the reaction on the 60.2 mg scale of **2d**, 54.9 mg (42.1%) of **3d** was obtained as a yellow oil. ^1^H NMR (400 MHz, CDCl_3_) δ 9.69 (s, 1H), 7.46 (d, *J* = 7.3 Hz, 2H), 7.05 (d, *J* = 7.3 Hz, 2H), 6.94 (s, 1H), 6.73 (s, 1H), 2.85 (t, *J* = 7.6 Hz, 2H), 2.77 (t, *J* = 7.5 Hz, 2H), 1.75–1.58 (m, 4H), 1.40–1.24 (m, 20H), 0.89–0.86 (m, 6H); ^13^C NMR (100 MHz, CDCl_3_) δ 182.81, 155.90, 152.59, 145.75, 143.64, 141.81, 137.13, 132.37, 128.18, 127.94, 125.80, 121.97, 118.53, 108.94, 31.77, 31.75, 31.17, 30.95, 30.64, 30.49, 29.19, 29.15, 29.12, 29.10, 29.06, 28.96, 28.88, 22.60, 14.07; IR (KBr): 2956, 2926, 2856 (C-H), 2228 (C≡N), 1661 (C=O) cm^−1^; HRMS–EI *m*/*z*: [M^+^ + Na] calcd for C_32_H_41_NOS_3_Na, 574.2244; found, 574.2243.

**Synthesis of 2'-[4-(*N*,*N*-dimethylamino)phenylsulfanyl]-5,5'-dioctyl-[3,3'-bithiophene]-2-carbaldehyde (3e):** The same procedure was used as for the synthesis of **3a**. From the reaction on the 66.3 mg scale of **2e**, 112.2 mg (83.2%) of **3e** was obtained as a yellow oil. ^1^H NMR (400 MHz, CDCl_3_) δ 9.72 (s, 1H), 7.16–7.14 (m, 2H), 6.92 (s, 1H), 6.74 (s, 1H), 6.59–6.56 (m, 2H), 2.93 (s, 6H), 2.85 (t, *J* = 7.6 Hz, 2H), 2.73 (t, *J* = 7.7 Hz, 2H), 1.72 (quint, *J* = 6.5 Hz, 2H), 1.65 (quint, *J* = 7.6 Hz, 2H), 1.41–1.28 (m, 20H), 0.89 (t, *J* = 10.8 Hz, 6H); ^13^C NMR (100 MHz, CDCl_3_) δ 183.48, 155.26, 150.00, 147.86, 145.27, 136.60, 135.78, 132.82, 132.29, 128.57, 127.25, 120.98, 112.59, 40.17, 31.74, 31.73, 31.16, 31.01, 30.68, 30.20, 29.18, 29.16, 29.09, 29.07, 29.01, 28.94, 22.56, 14.02; IR (KBr): 2954, 2924, 2851 (C-H), 1659 (C=O) cm^−1^; HRMS–EI *m*/*z*: [M^+^ + Na] calcd for C_33_H_47_NOS_3_Na, 592.2709; found, 592.2712.

**Synthesis of 2'-(1-naphthylsulfanyl)-5,5'-dioctyl-[3,3'-bithiophene]-2-carbaldehyde (3f):** The same procedure was used as for the synthesis of **3a**. From the reaction on the 72.4 mg scale of **2f**, 84.7 mg (60.5%) of **3f** was obtained as a yellow oil. ^1^H NMR (400 MHz, CDCl_3_) δ 9.76 (s, 1H), 8.22–8.19 (m, 1H), 7.84–7.82 (m, 1H), 7.71 (d, *J* = 8.2 Hz, 1H), 7.51–7.49 (m, 2H), 7.34 (t, *J* = 7.56 Hz, 1H), 7.25 (d, *J* = 7.21 Hz, 1H), 6.87 (s, 1H), 6.84 (s, 1H), 2.81 (t, *J* = 7.6 Hz, 2H), 2.75 (t, *J* = 7.5 Hz, 2H), 1.70 (quint, *J* = 7.3 Hz, 2H), 1.59 (quint, *J* = 7.6 Hz, 2H), 1.39–1.25 (m, 22H), 0.90 (t, *J* = 5.8 Hz, 6H); ^13^C NMR (100 MHz, CDCl_3_) δ 183.23, 155.54, 150.30, 144.76, 139.41, 136.91, 134.74, 133.70, 131.40, 128.35, 128.34, 127.71, 127.42, 127.12, 126.73, 126.39, 126.26, 125.56, 124.25, 31.76, 31.16, 30.85, 30.60, 30.36, 29.19, 29.12, 29.06, 29.04, 28.88, 22.60, 14.08; IR (KBr): 2956, 2926, 2855 (C-H), 1661 (C=O) cm^−1^; HRMS–EI *m*/*z*: [M^+^ + Na] calcd for C_35_H_44_OS_3_Na, 599.2443; found, 599.2447.

**Synthesis of 2'-(9-anthrylsulfanyl)-5,5'-dioctyl-[3,3'-bithiophene]-2-carbaldehyde (3g):** The same procedure was used as for the synthesis of **3a**. From the reaction on the 87.4 mg scale of **2g**, 85.8 mg (56.4%) of **3g** was obtained as a yellow oil. ^1^H NMR (400 MHz, CDCl_3_) δ 9.60 (s, 1H), 8.62 (d, *J* = 9.4 Hz, 2H), 8.47 (s, 1H), 7.98 (d, *J* = 9.1 Hz, 2H), 7.51–7.45 (m, 4H), 6.91 (s, 1H), 6.60 ( s, 1H), 2.85 (t, *J* = 7.6 Hz, 2H), 2.54 (t, *J* = 7.7 Hz, 2H), 1.75 (quint, *J* = 3.0 Hz, 2H), 1.48–1.20 (m, 22H), 0.89 (dd, *J* = 6.4 Hz, 12.8 Hz, 6H); ^13^C NMR (100 MHz, CDCl_3_) δ 183.32, 155.58, 147.19, 145.39, 137.02, 135.14, 134.08, 133.82, 132.41, 131.76, 130.06, 128.82, 128.76, 128.14, 127.18, 126.94, 126.57, 125.41, 31.82, 31.74, 31.09, 31.06, 30.77, 30.10, 29.68, 29.27, 29.18, 29.13, 29.09, 20.08, 28.99, 22.63, 22.58, 14.07, 14.06; IR (KBr): 2956, 2926, 2856 (C-H), 1649 (C=O) cm^−1^; HRMS–EI *m*/*z*: [M^+^ + Na] calcd for C_39_H_46_OS_3_Na, 649.2613; found, 649.2603.

**Synthesis of 2'-[4-(*N*,*N*-diphenylamino)phenylsulfanyl]-5,5'-dioctyl-[3,3'-bithiophene]-2-carbaldehyde (3h):** The same procedure was used as for the synthesis of **3a**. From the reaction on the 100.1 mg scale of **2h**, 127.4 mg (75.6%) of **3h** was obtained as a yellow oil. ^1^H NMR (400 MHz, CDCl_3_) δ 9.65 (s, 1H), 7.26–7.22 (m, 3H), 7.06–7.02 (m, 6H), 7.00–6.99 (m, 1H), 6.98–6.97 (m, 2H), 6.91–6.88 (m, 2H) 6.88 (s, 1H), 6.81 (s, 1H), 2.85–2.77 (m, 4H), 1.73–1.65 (m, 4H), 1.39–1.26 (m, 20H), 0.90 (td, *J* = 6.4 Hz, 3.2 Hz, 6H); ^13^C NMR (100 MHz, CDCl_3_) δ 183.28, 155.41, 149.67, 147.28, 146.82, 144.92, 138.63, 136.89, 129.94, 129.55, 129.25, 128.50, 128.27, 127.69, 124.47, 123.63, 123.12, 31.80, 31.78, 31.23, 31.04, 30.75, 30.36, 29.23, 29.21, 29.14, 29.09, 29.00, 22.62, 14.10. IR (KBr): 2956, 2926, 2855 (C-H), 1661 (C=O) cm^−1^; HRMS–EI *m*/*z*: [M^+^ + Na] calcd for C_43_H_51_NOS_3_ Na, 716.3037; found, 716.3025.

**Synthesis of 2'-(1-pyrenylsulfanyl)-5,5'-dioctyl-[3,3'-bithiophene]-2-carbaldehyde (3i):** The same procedure was used as for the synthesis of **3a** except for two differences. One is that *t*-BuLi (2.6 equiv) was employed for the metal–halogen exchange, and another one is that the temperature for the ring-opening reaction of **1** was set to 0 °C for 3h. From the reaction on the 87.7 mg (1.4 equiv) scale of **2i**, 69.1 mg (47.7%) of **3i** and a side product, 1-pyrenecarboxaldehyde (27.8 mg, 42.0%) were generated. **3i** was obtained as a light yellow solid, mp 66–68 °C; ^1^H NMR (400 MHz, CDCl_3_) δ 9.79 (s, 1H), 8.43 (d, *J* = 9.2 Hz, 1H), 8.18–8.16 (m, 2H), 8.08–7.95 (m, 5H), 7.80 (d, *J* = 8.1 Hz, 1H), 6.84 (s, 1H), 6.75 (s, 1H), 2.77 (t, *J* = 7.6 Hz, 2H), 2.63 (t, *J* = 7.6 Hz, 2H), 1.70–1.63 (m, 2H), 1.47–1.13 (m, 22H), 0.88 (t, *J* = 6.8 Hz, 3H), 0.85 (t, *J* = 7.0 Hz, 3H); ^13^C NMR (100 MHz, CDCl_3_) δ 183.27, 155.54, 149.92, 144.82, 138.90, 136.93, 131.40, 131.15, 130.71, 130.36, 129.58, 128.27, 128.12, 127.92, 127.62, 127.54, 127.00, 126.12, 125.36, 125.28, 124.94, 124.86, 124.18, 123.54, 31.75, 31.70, 31.15, 30,74, 30.52, 30.34, 29.17, 29.10, 29.03, 29.01, 28.84, 22.59, 22.55, 14.06, 14.04; IR (KBr): 2956, 2926, 2854 (C-H), 1653 (C=O) cm^−1^; HRMS–EI *m*/*z*: [M^+^ + Na] calcd for C_41_H_46_OS_3_Na, 673.2611; found, 673.2603.

**Synthesis of 2-butyl-2'-(1-pyrenylsulfanyl)-5,5'-dioctyl-3,3'-bithiophene (4):** To a solution of **2i** (93.7 mg, 0.33 mmol, 1.4 equiv) in dry THF (10 mL), *n*-BuLi (2.39 M in hexane, 0.13 mL, 0.31 mmol, 1.3 equiv) was added dropwise at −78 °C. After slowly warming to −78 °C for 2 h, a solution of **1** (100.1 mg, 0.24 mmol, 1.0 equiv) in dry THF (10 mL) was added dropwise. The reaction mixture was slowly warmed to 0 °C for 3 h, then cooled back to −78 °C. Dry DMF (0.05 mL, 0.48 mmol, 2.0 equiv) was added dropwise, and the reaction mixture was slowly warmed to ambient temperature overnight. After quenching with H_2_O (30 mL), the reaction mixture was extracted with CHCl_3_ (2 × 30 mL) and then washed with H_2_O (30 mL). After drying over MgSO_4_, the solvent was removed in vacuum. The residue was purified by column chromatography on silica gel with petrol ether (60–90 °C)/chloroform (1:4, v/v) as eluents, 72.6 mg (45%) of **4** was obtained as a brown oil, and **5** (24.5 mg, 35%) was also obtained. For **4**, ^1^H NMR (400 MHz, CDCl_3_) δ 8.49 (d, *J* = 9.2 Hz, 1H), 8.18–8.15 (dd, *J* = 3.6, 7.7 Hz, 2H), 8.07 (d, 1H), 8.04–7.95 (m, 4H), 7.80 (d, *J* = 8.1 Hz, 1H), 6.77 (s, 1H), 6.58 (s, 1H), 2.78 (t, *J* = 7.5 Hz, 2H), 2.69 (t, *J* = 7.5 Hz, 2H), 2.60 (t, *J* = 7.6 Hz, 2H), 1.67 (qt, *J* = 7.4 Hz, 2H), 1.51–1.42 (m, 4H), 1.32–1.10 (m, 22H), 0.89 (t, *J* = 6.8 Hz, 3H), 0.85 (t, *J* = 7.0 Hz, 3H), 0.80 (t, *J* = 7.4 Hz, 3H); ^13^C NMR (100 MHz, CDCl_3_) δ 149.28, 143.27, 141.10, 139.92, 133.08, 131.48, 131.34, 130.95, 129.96, 129.25, 127.62, 127.43, 127.36, 127.21, 126.07, 126.02, 125.16, 125.11, 124.95, 124.55, 124.45, 123.89, 34.04, 31.83, 31.81, 31.27, 31.22, 30.53, 29.92, 29.71, 29.28, 29.21, 29.15, 29.06, 28.52, 22.66, 22.62, 22.39, 14.11, 13.79; IR (KBr): 3435 (Ar-H), 2956, 2924, 2855 (C-H) cm^−1^; HRMS–EI *m*/*z*: [M]^+^ calcd for C_44_H_54_S_3_, 678.3392; found, 678.3382.

## Supporting Information

File 1 Characterization data and NMR spectra of all compounds including the X-ray structure determination of **3i**.

File 2 X-ray crystallographic data file of **3i**.
