# Supplementary material for: Ring-opening reaction of 2,5-dioctyldithieno[2,3-b:3',2'-d]thiophene in the presence of aryllithium reagents
Source: Beilstein J Org Chem. 2013 Apr 19;9:767–74. doi: 10.3762/bjoc.9.87 (PMC3678665; doi:10.3762/bjoc.9.87)
Supplement: File 1 — Characterization data and NMR spectra of all compounds including the X-ray structure determination of 3i. [file Beilstein_J_Org_Chem-09-767-s001.pdf]

Supporting Information

for

Ring-opening reaction of 2,5-dioctyldithieno[2,3-*b*:3',2'-*d*]thiophene in the presence of aryllithium reagents

Hao Zhong[1], Jianwu Shi[1], Jianxun Kang[2], Shaomin Wang[2], Xinming Liu[1] and Hua Wang*[1]

Address: [1]Key Lab for Special Functional Materials of Ministry of Education, Henan University, Kaifeng, 475004, China and [2]Department of Chemistry, Zhengzhou University, Zhengzhou, 450001, China

Email: Hua Wang* - hwang@henu.edu.cn

* Corresponding author

Characterization data and NMR spectra of all compounds, including the X-ray structure determination of 3i

Table of contents

1. General (S2)
2. Reference (S2)
3. NMR and HRMS spectra from **3a** to **3i** and **5** (S3–S17)
4. X-ray crystallographic data for **3i** (S18–S24)

1. General

Tetrahydrofuran (THF) was freshly distilled from sodium/benzophenone under an argon atmosphere prior to use. 2,5-dioctyldithieno[2,3-*b*:3',2'-*d*]thiophene (**1**) was obtained according to the literature method [S1]. **2a**–**2e** were commercially available. **2f**–**2i** were obtained by regular bromination methods from correlated aryl compounds. Concentrations of *n*-BuLi (in hexane) and *t*-BuLi (in pentane) were determined by titration with *N*-pivaloyl-*o*-toluidine [S2]. Column chromatography was carried out on silica gel (300–400 mesh). Analytical thin-layer chromatography was performed on glass plates of Silica Gel GF_{254} with detection by UV. Standard techniques for synthesis under inert atmosphere, by using Schlenk glassware equipped with an 8 mm PTFE vacuum stop-cock, were employed. NMR spectra were obtained by using chloroform-*d* ($CDCl_3$) as a solvent. The chemical shift references were as follows: (^1H) $CDCl_3$, 7.26 ppm ($CHCl_3$); (^{13}C) $CDCl_3$, 77.00 ppm ($CDCl_3$). HRMS spectra were recorded on a mass spectrometer equipped with TOF (EI^+). IR spectra were obtained by using an FT-IR instrument, equipped with an ATR sampling accessory. Melting points were determined with a Melt-Temp apparatus and were uncorrected.

The X-ray crystallographic analyses were performed by using crystals of compound **3i** with the size 0.44 × 0.35 × 0.11 mm. The intensity data were collected with the ω scan mode (207 K) on a diffractometer with a CCD detector by using the Mo Kα radiation (λ = 0.71073 Å). The data were corrected for Lorentz and polarization effects and absorption corrections were performed by using the SADABS program [S3]. The crystal structure was solved by using the SHELXTL program and refined by using full matrix least squares [S4]. The positions of hydrogen atoms were calculated theoretically and included in the final cycles of refinement in a riding model along with the attached carbons. Further details are in the deposited CIFs. Slow evaporation of a solution of **3i** in $CHCl_3/CH_3OH$ (1:5 v/v) was employed for growing single crystals.

2. References

[S1]. Miyasaka, M.; Rajca, A. *J. Org. Chem.* **2006**, *71*, 3264–3266.
[S2]. Suffert, J. *J. Org. Chem.* **1989**, *54*, 509–510.
[S3]. Sheldrick, G. M. *SADABS*; University of Göttingen: Germany, **1996**.
[S4]. Sheldrick, G. M. *SHELXTL*, *version* 5.1; Bruker Analytical X-ray Systems: Madison,WI, **1997**.

3. NMR and HRMS spectra from 3a to 3i and 5

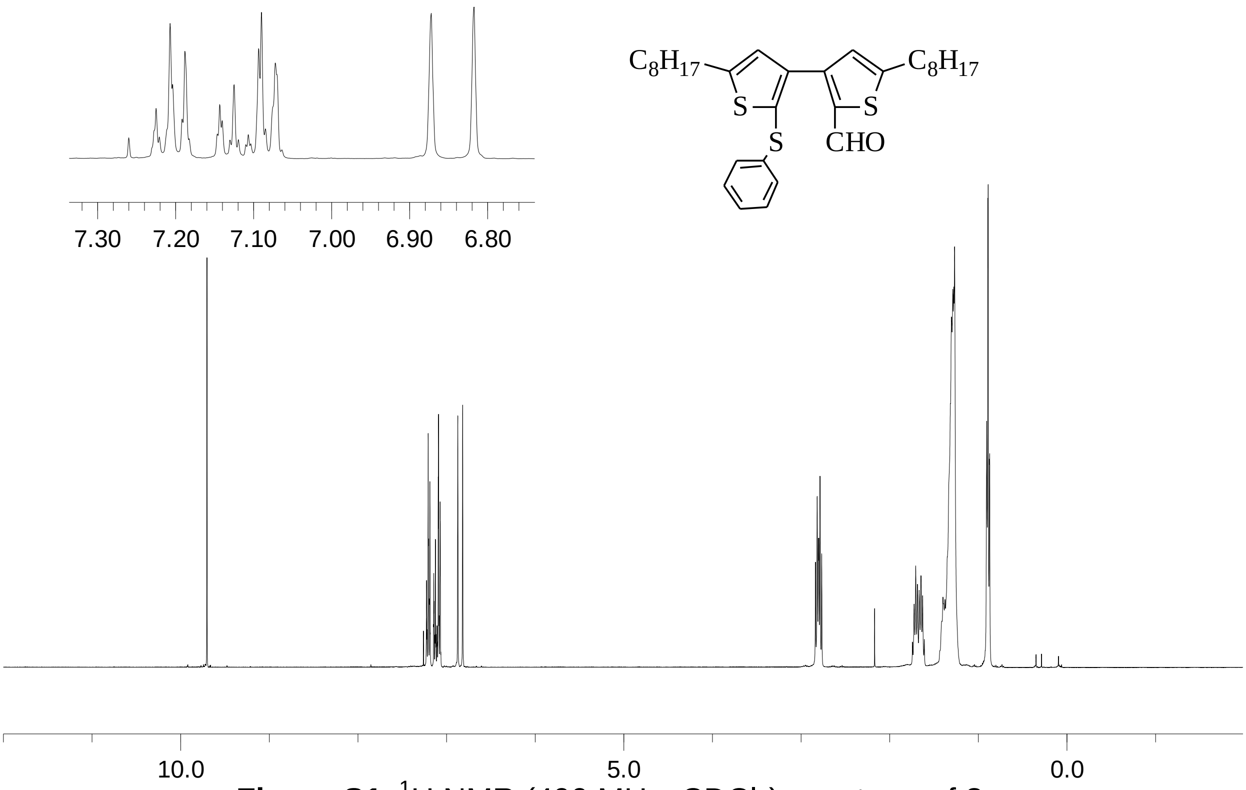

Figure S1: ^{1}H NMR (400 MHz, $CDCl_3$) spectrum of **3a**.

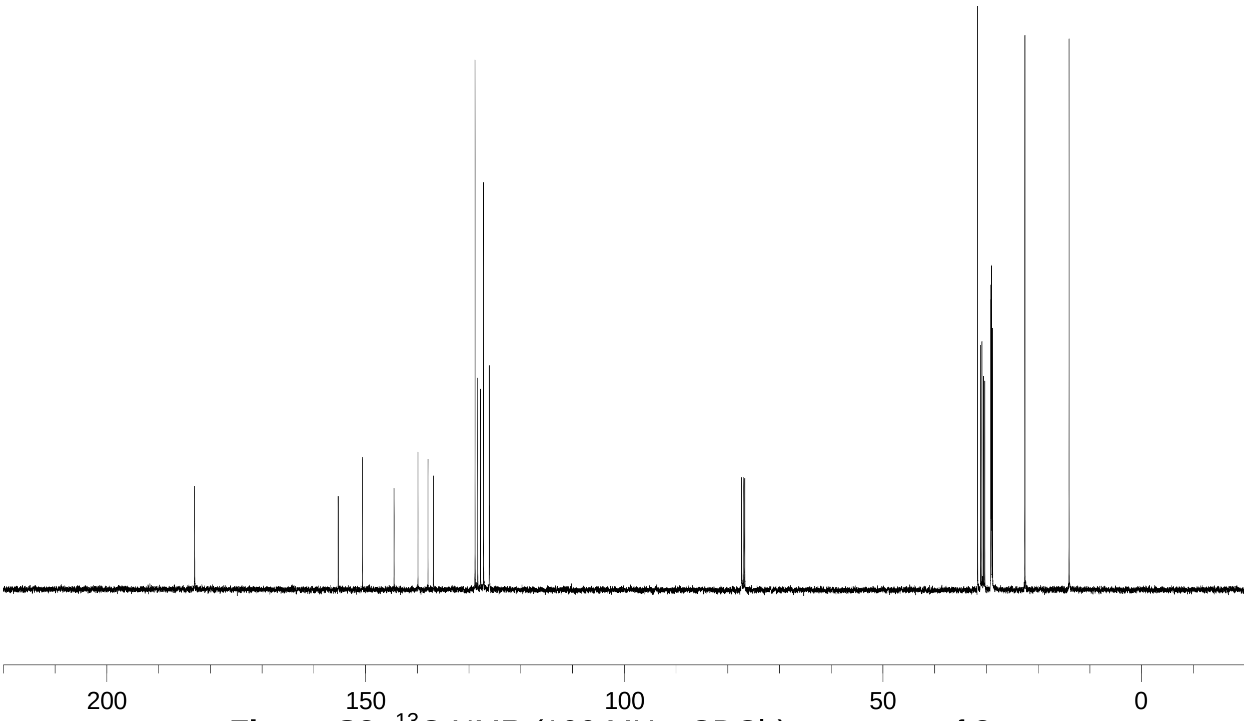

Figure S2: ^{13}C NMR (100 MHz, $CDCl_3$) spectrum of **3a**.

Instrument: IonSpec 4.7 Tesla FTMS

Card Serial Number : WI12 0429

Sample Serial Number: zh-2-71-col

Operator : HuaQin   Date: 2012/03/22

Operation Mode: MALDI/DHB

Elemental Composition Search Report:

Target Mass:

Target m/z = 549.2293 ± 0.003
Charge = +1

Possible Elements:

Element:	Exact Mass:	Min:	Max:
C	12.000000	0	100
H	1.007825	0	100
O	15.994915	0	3
S	31.972071	0	3
Na	22.989770	0	1

Additional Search Restrictions:

DBE Limit Mode = Both Integer and Half-Integer
Minimum DBE = 0

Search Results:

Number of Hits = 3

m/z	Delta m/z	DBE	Formula
549.22900	0.00030	11.0	$C_{31}H_{42}OS_3Na^{+1}$
549.22803	0.00127	18.5	$C_{36}H_{37}OS_2^{+1}$
549.23140	-0.00210	13.5	$C_{33}H_{41}OS_3^{+1}$

Figure S3: HRMS data of **3a**.

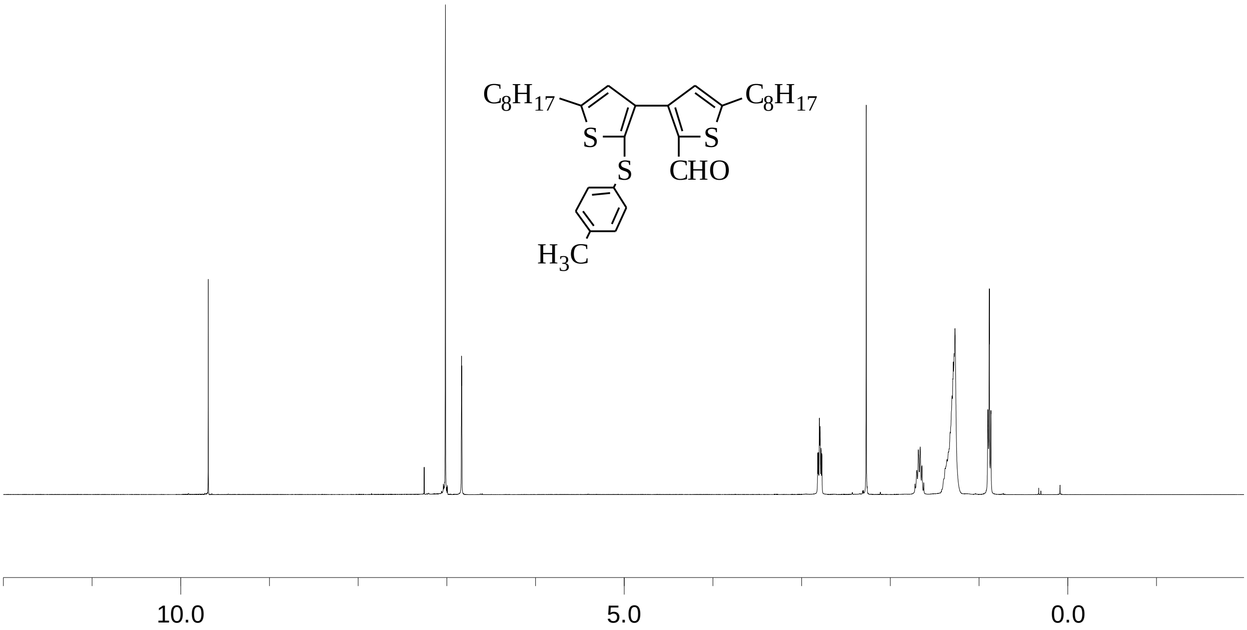

Figure S4: ^{1}H NMR (400 MHz, $CDCl_3$) spectrum of **3b**.

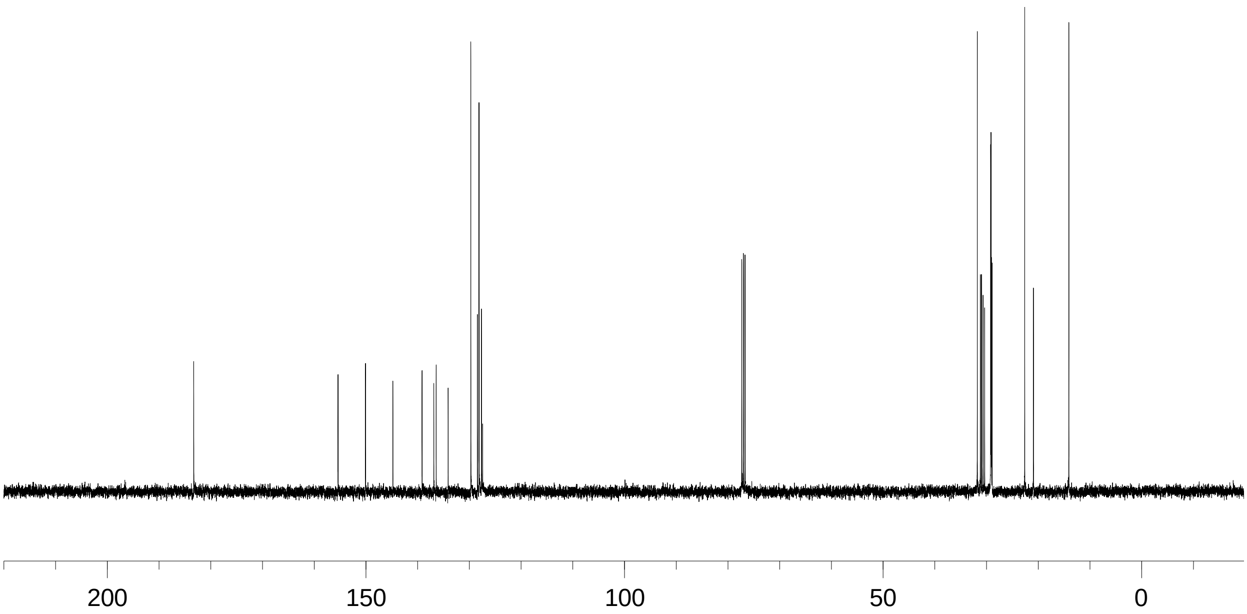

Figure S5: ^{13}C NMR (100 MHz, $CDCl_3$) spectrum of **3b**.

Instrument: IonSpec 4.7 Tesla FTMS

Card Serial Number : WI12 0425

Sample Serial Number: ZH-3-7-col2

Operator : HuaQin Date: 2012/03/22

Operation Mode: MALDI/DHB

Elemental Composition Search Report:

Target Mass:
Target m/z = 563.2430 ± 0.003
Charge = +1

Possible Elements:

Element:	Exact Mass:	Min:	Max:
C	12.000000	0	100
H	1.007825	0	100
O	15.994915	0	5
S	31.972071	0	5
Na	22.989770	0	1

Additional Search Restrictions:
DBE Limit Mode = Both Integer and Half-Integer
Minimum DBE = 0

Search Results:
Number of Hits = 4

m/z	Delta m/z	DBE	Formula
563.24368	-0.00068	18.5	$C_{37}H_{39}OS_2^{+1}$
563.24465	-0.00165	11.0	$C_{32}H_{44}OS_3Na^{+1}$
563.24128	0.00172	16.0	$C_{35}H_{40}OS_2Na^{+1}$
563.24031	0.00269	23.5	$C_{40}H_{35}OS^{+1}$

Figure S6: HRMS data of **3b**.

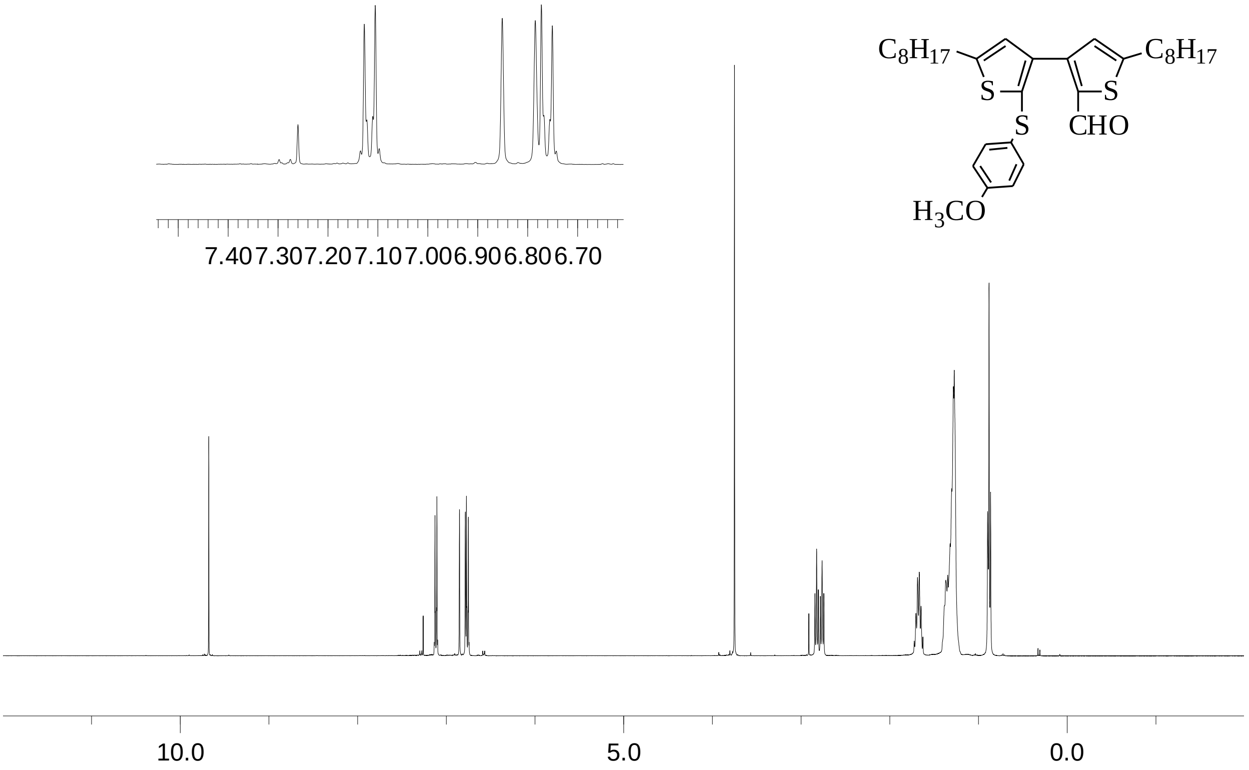

Figure S7: ^{1}H NMR (400 MHz, $CDCl_3$) spectrum of **3c**.

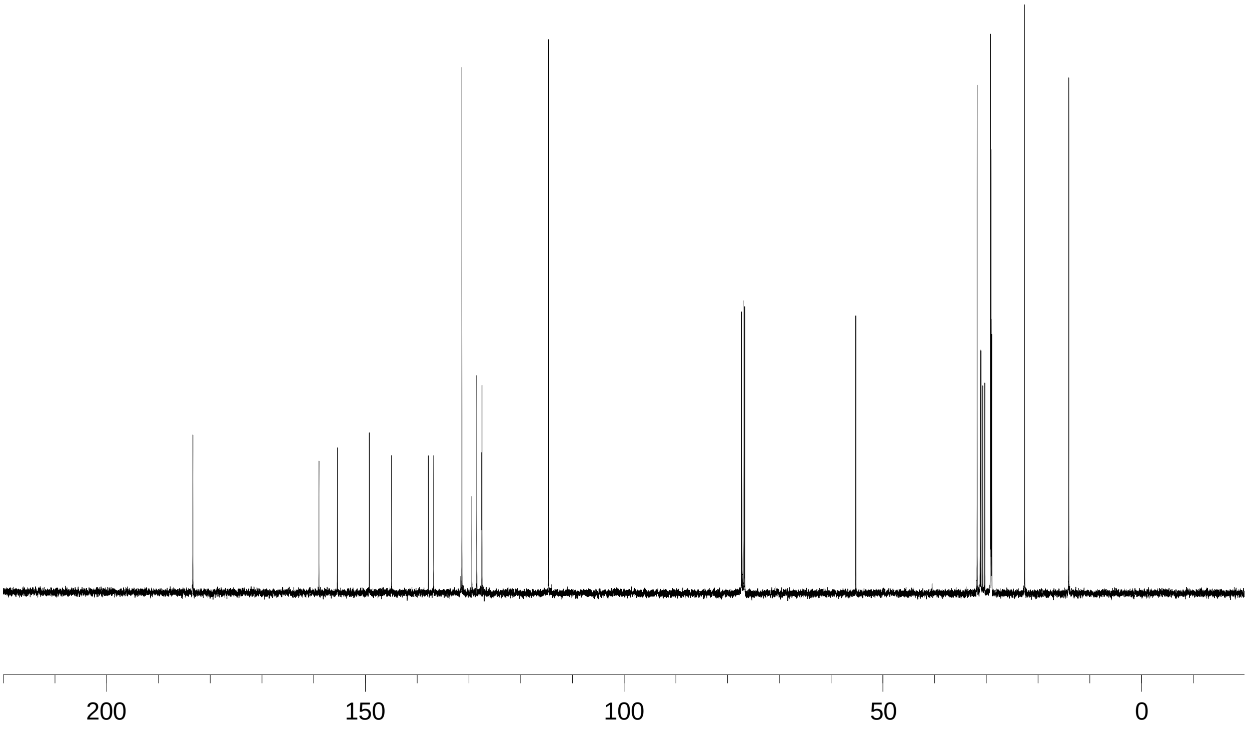

Figure S8: ^{13}C NMR (100 MHz, $CDCl_3$) spectrum of **3c**.

Instrument: IonSpec 4.7 Tesla FTMS

Card Serial Number : WI12 0432

Sample Serial Number: ZH-3-9-col3

Operator : HuaQin Date: 2012/03/22

Operation Mode: MALDI/DHB

Elemental Composition Search Report:

Target Mass:

Target m/z = 579.2395 ± 0.003
Charge = +1

Possible Elements:

Element:	Exact Mass:	Min:	Max:
C	12.000000	0	100
H	1.007825	0	100
O	15.994915	0	3
S	31.972071	0	3
Na	22.989770	0	1

Additional Search Restrictions:

DBE Limit Mode = Both Integer and Half-Integer
Minimum DBE = 0

Search Results:

Number of Hits = 3

m/z	Delta m/z	DBE	Formula
579.23956	-0.00006	11.0	$C_{32}H_{44}O_2S_3Na^{+1}$
579.23860	0.00090	18.5	$C_{37}H_{39}O_2S_2^{+1}$
579.24197	-0.00247	13.5	$C_{34}H_{43}O_2S_3^{+1}$

Figure S9: HRMS data of **3c**.

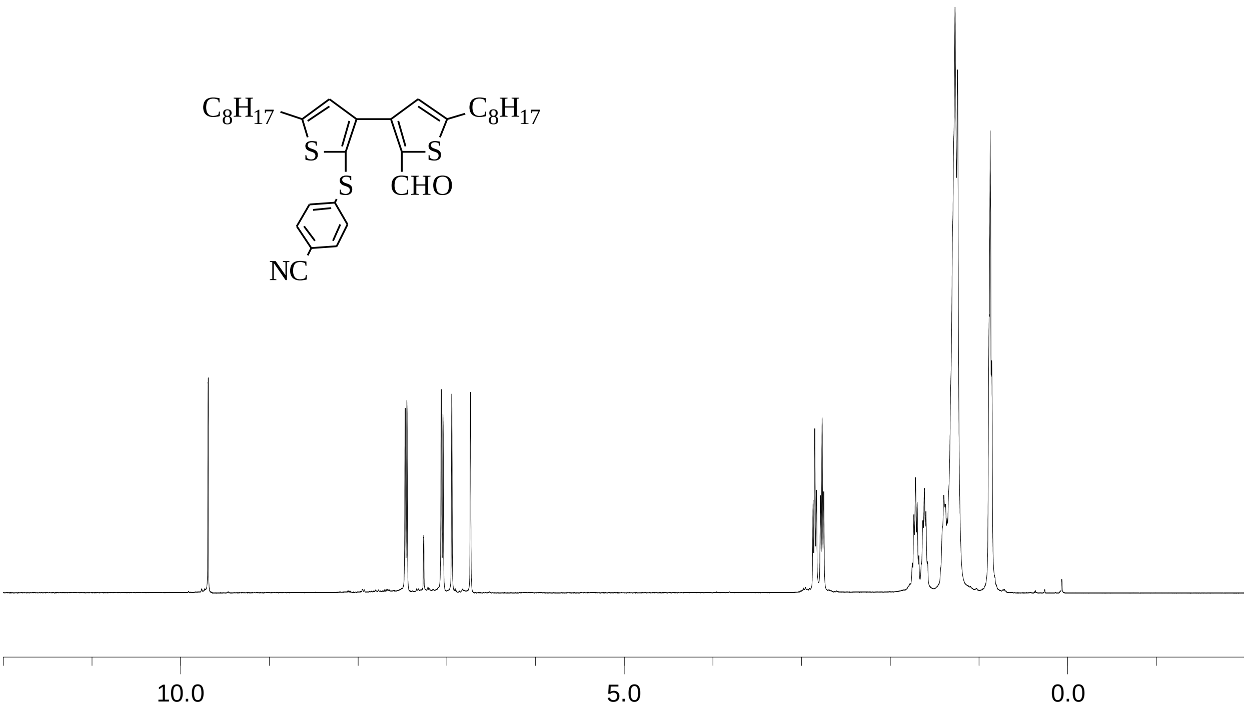

Figure S10: ^{1}H NMR (400 MHz, $CDCl_3$) spectrum of **3d**.

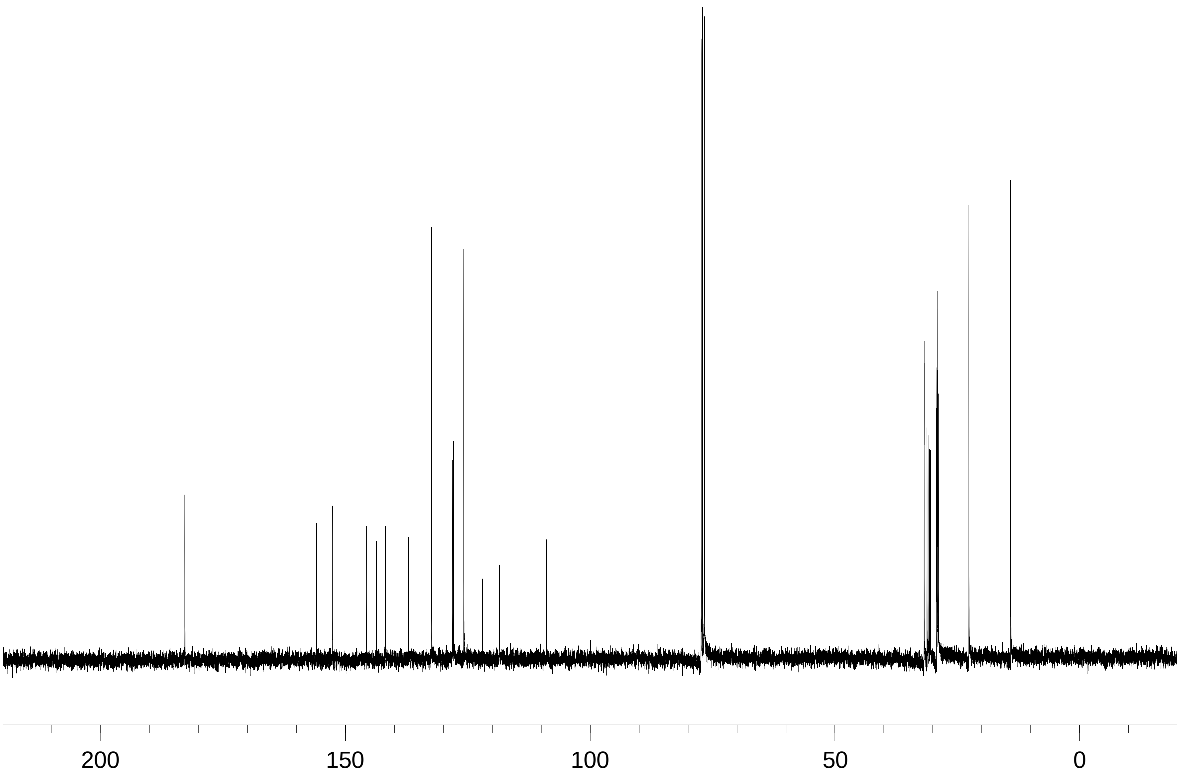

Figure S11: ^{13}C NMR (100 MHz, $CDCl_3$) spectrum of **3d**.

Instrument: IonSpec 4.7 Tesla FTMS

Card Serial Number : WI12 0654

Sample Serial Number: zh-3-15-col

Operator : HuaQin Date: 2012/05/15

Operation Mode: MALDI/DHB

Elemental Composition Search Report:

Target Mass:

Target m/z = 574.2244 ± 0.002

Charge = +1

Possible Elements:

Element	Exact Mass:	Min:	Max:
C	12.000000	0	100
H	1.007825	0	100
N	14.003074	0	3
O	15.994915	0	3
S	31.972071	0	3
Na	22.989770	0	1

Additional Search Restrictions:

DBE Limit Mode = Both Integer and Half-Integer

Minimum DBE = 0

Search Results:

Number of Hits = 7

m/z	Delta m/z	DBE	Formula
574.22425	0.00015	13.0	$C_{32}H_{41}NOS_3Na^{+1}$
574.22509	-0.00069	26.0	$C_{39}H_{30}N_2O_3^{+1}$
574.22537	-0.00097	28.0	$C_{40}H_{29}N_3Na^{+1}$
574.22328	0.00112	20.5	$C_{37}H_{36}NOS_2^{+1}$
574.22606	-0.00166	18.5	$C_{34}H_{35}N_2O_3SNa^{+1}$
574.22269	0.00171	23.5	$C_{37}H_{31}N_2O_3Na^{+1}$
574.22263	0.00177	11.5	$C_{29}H_{40}N_3O_3S_3^{+1}$

Figure S12: HRMS data of **3d**.

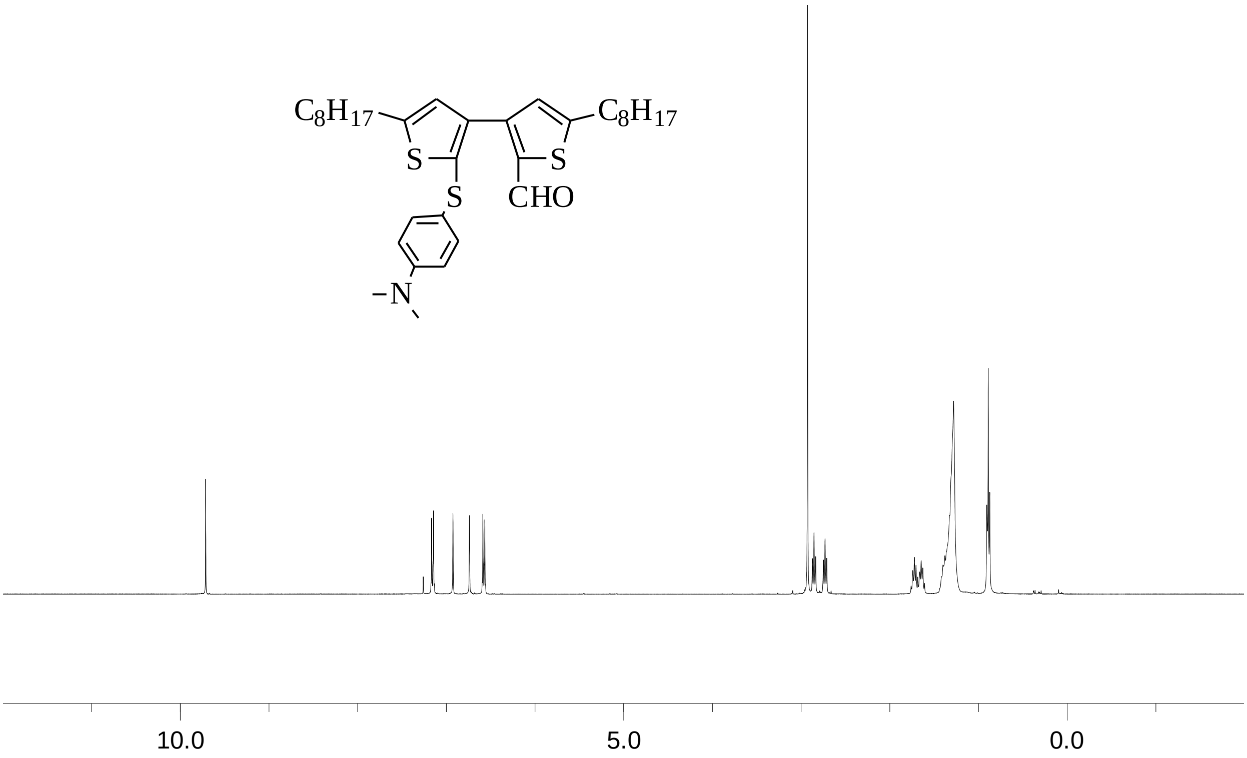

Figure S13: ^{1}H NMR (400 MHz, $CDCl_3$) spectrum of **3e**.

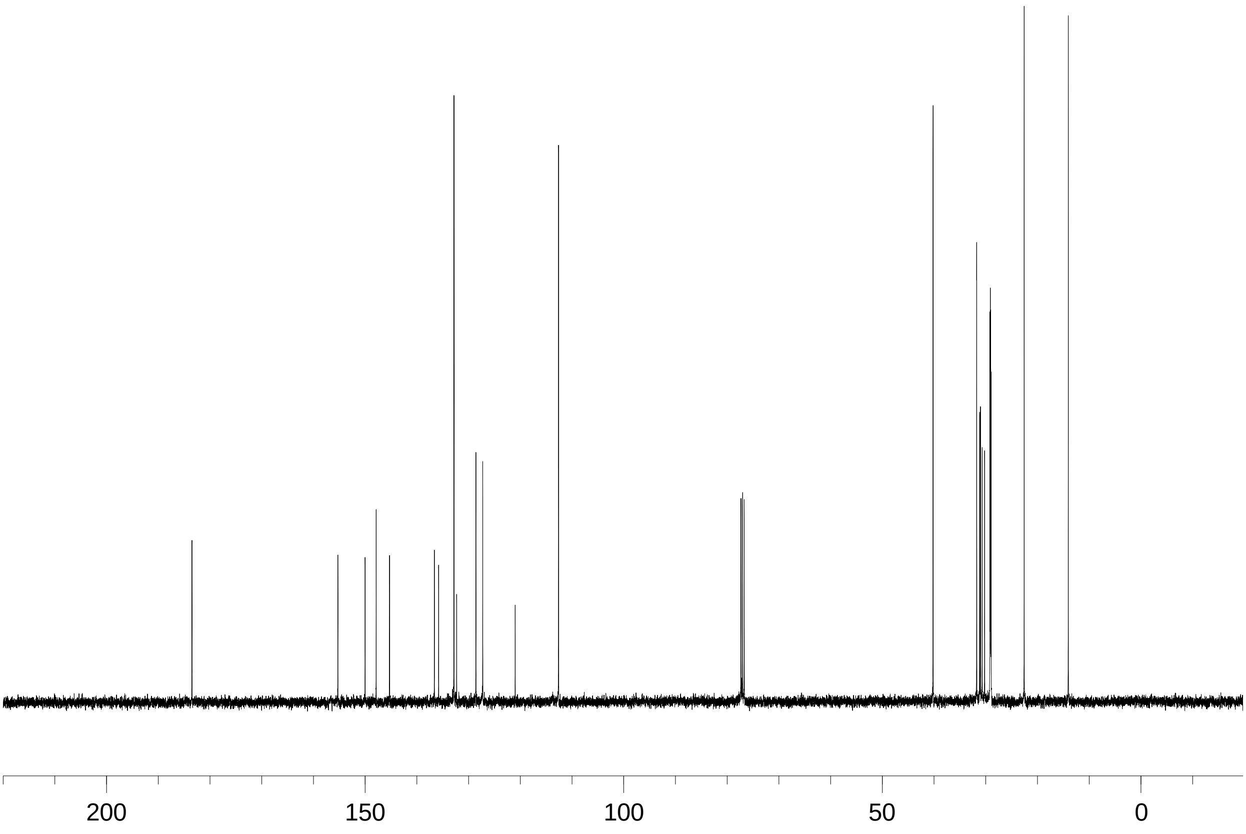

Figure S14: ^{13}C NMR (100 MHz, $CDCl_3$) spectrum of **3e**.

Instrument: IonSpec 4.7 Tesla FTMS

Card Serial Number : WI12 0426

Sample Serial Number: ZH-3-11-col

Operator : HuaQin Date: 2012/03/22

Operation Mode: MALDI/DHB

Elemental Composition Search Report:

Target Mass:
Target m/z = 592.2709 ± 0.003
Charge = +1

Possible Elements:

Element:	Exact Mass:	Min:	Max:
C	12.000000	0	100
H	1.007825	0	100
N	14.003074	0	3
O	15.994915	0	3
S	31.972071	0	3
Na	22.989770	1	1

Additional Search Restrictions:
DBE Limit Mode = Both Integer and Half-Integer
Minimum DBE = 0

Search Results:
Number of Hits = 5

m/z	Delta m/z	DBE	Formula
592.27120	-0.00030	11.0	$C_{33}H_{47}NOS_3Na^{+1}$
592.26964	0.00126	21.5	$C_{38}H_{37}N_2O_3Na^{+1}$
592.27232	-0.00142	26.0	$C_{41}H_{35}N_3Na^{+1}$
592.27301	-0.00211	16.5	$C_{35}H_{41}N_2O_3SNa^{+1}$
592.27366	-0.00276	25.5	$C_{43}H_{37}ONa^{+1}$

Figure S15: HRMS data of **3e**.

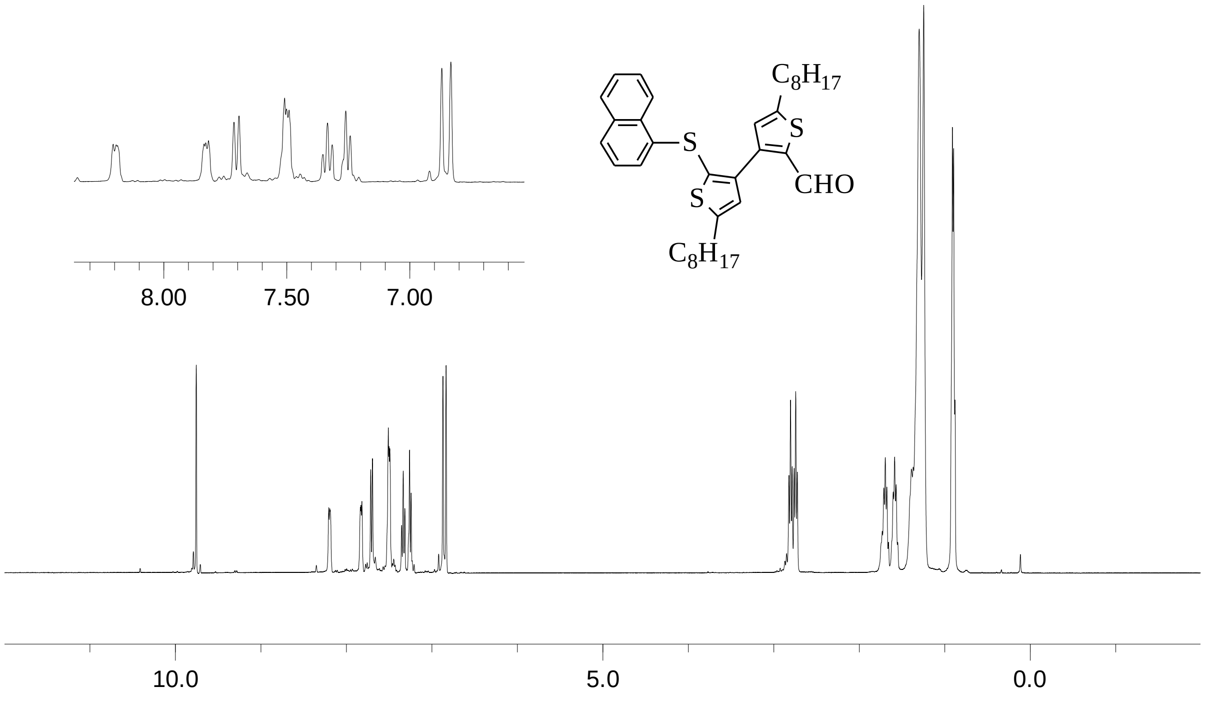

Figure S16: ^{1}H NMR (400 MHz, $CDCl_3$) spectrum of **3f**.

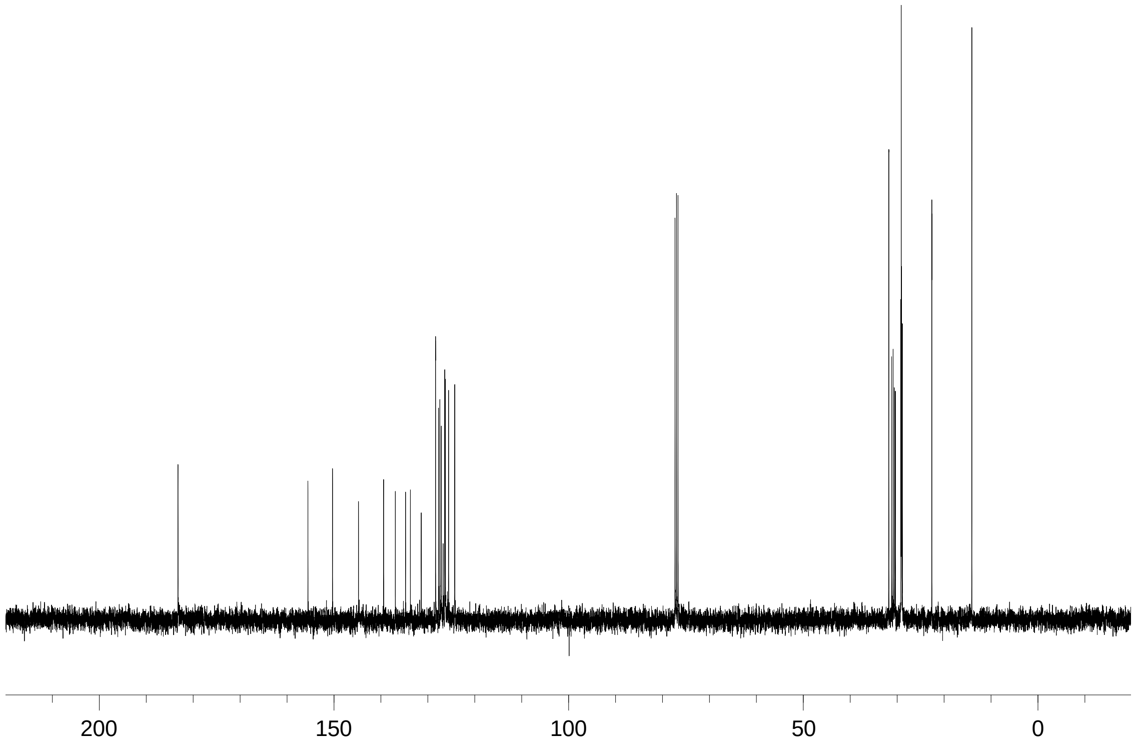

Figure S17: ^{13}C NMR (100 MHz, $CDCl_3$) spectrum of **3f**.

Instrument: IonSpec 4.7 Tesla FTMS

Card Serial Number : WI12 0430

Sample Serial Number: zh-2-73-col2

Operator : HuaQin Date: 2012/03/22

Operation Mode: MALDI/DHB

Elemental Composition Search Report:

Target Mass:

Target m/z = 599.2443 ± 0.003
Charge = +1

Possible Elements:

Element:	Exact Mass:	Min:	Max:
C	12.000000	0	100
H	1.007825	0	100
O	15.994915	0	3
S	31.972071	0	3
Na	22.989770	0	1

Additional Search Restrictions:

DBE Limit Mode = Both Integer and Half-Integer*
Minimum DBE = 0

Search Results:

Number of Hits = 3

m/z	Delta m/z	DBE	Formula
599.24465	-0.00035	14.0	$C_{35}H_{44}OS_3Na^{+1}$
599.24368	0.00062	21.5	$C_{40}H_{39}OS_2^{+1}$
599.24705	-0.00275	16.5	$C_{37}H_{43}OS_3^{+1}$

Figure S18: HRMS data of **3f**.

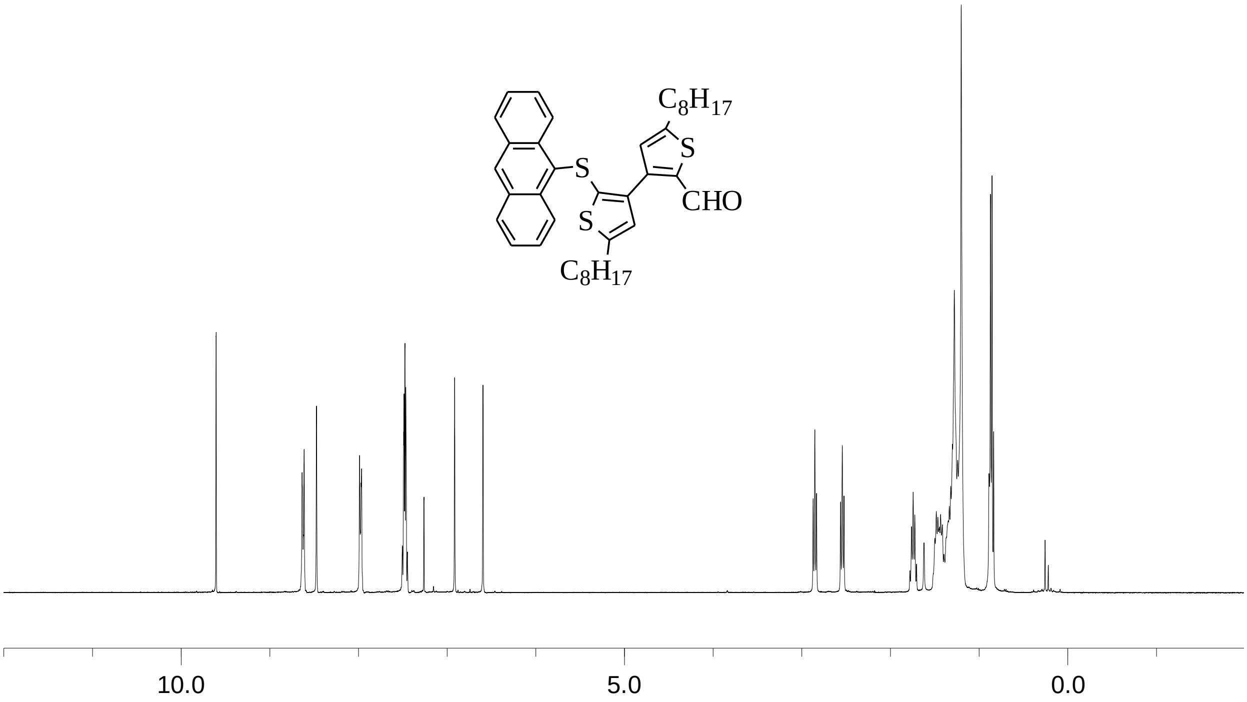

Figure S19: ^{1}H NMR (400 MHz, $CDCl_3$) spectrum of **3g**.

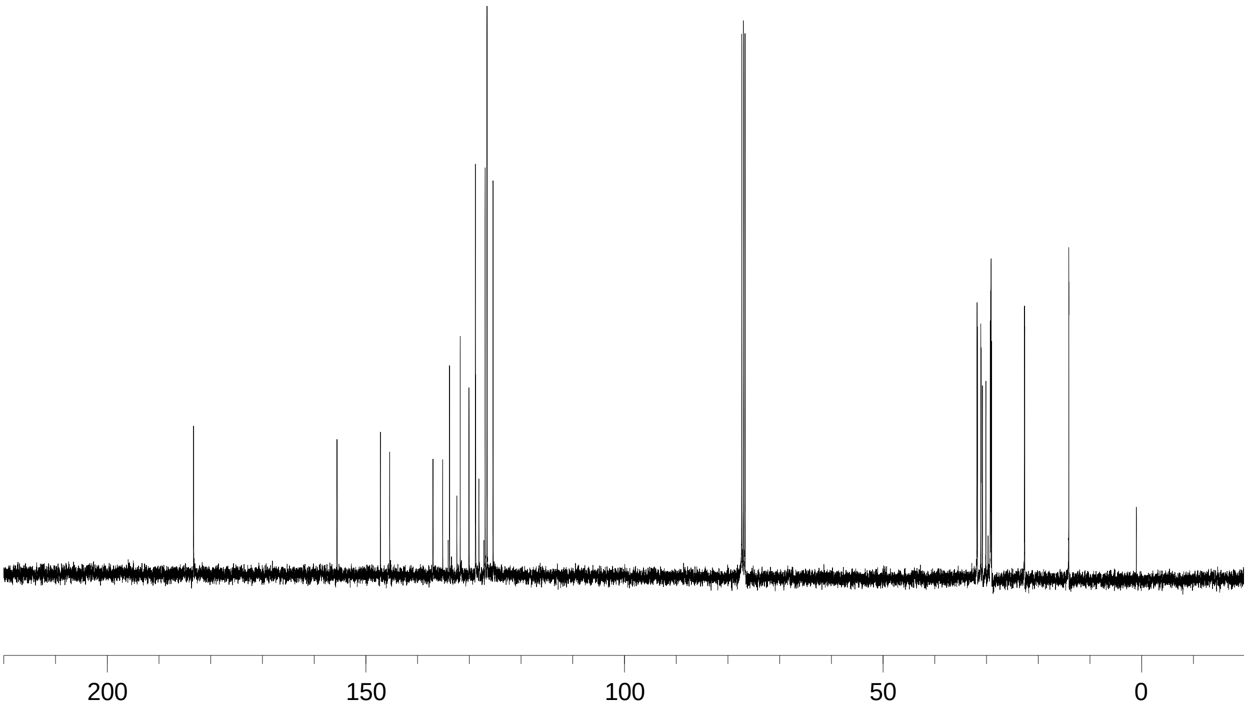

Figure S20: ^{13}C NMR (100 MHz, $CDCl_3$) spectrum of **3g**.

Instrument: IonSpec 4.7 Tesla FTMS

Card Serial Number : WI12 0431

Sample Serial Number: zh-2-67-col3

Operator : HuaQin Date: 2012/03/22

Operation Mode: MALDI/DHB

Elemental Composition Search Report:

Target Mass:

Target m/z = 649.2613 ± 0.003
Charge = +1

Possible Elements:

Element:	Exact Mass:	Min:	Max:
C	12.000000	0	100
H	1.007825	0	100
O	15.994915	0	3
S	31.972071	0	3
Na	22.989770	0	1

Additional Search Restrictions:

DBE Limit Mode = Both Integer and Half-Integer
Minimum DBE = 0

Search Results:

Number of Hits = 3

m/z	Delta m/z	DBE	Formula
649.26030	0.00100	17.0	$C_{39}H_{46}OS_3Na^{+1}$
649.26270	-0.00140	19.5	$C_{41}H_{45}OS_3^{+1}$
649.25933	0.00197	24.5	$C_{44}H_{41}OS_2^{+1}$

Figure S21: HRMS data of **3g**.

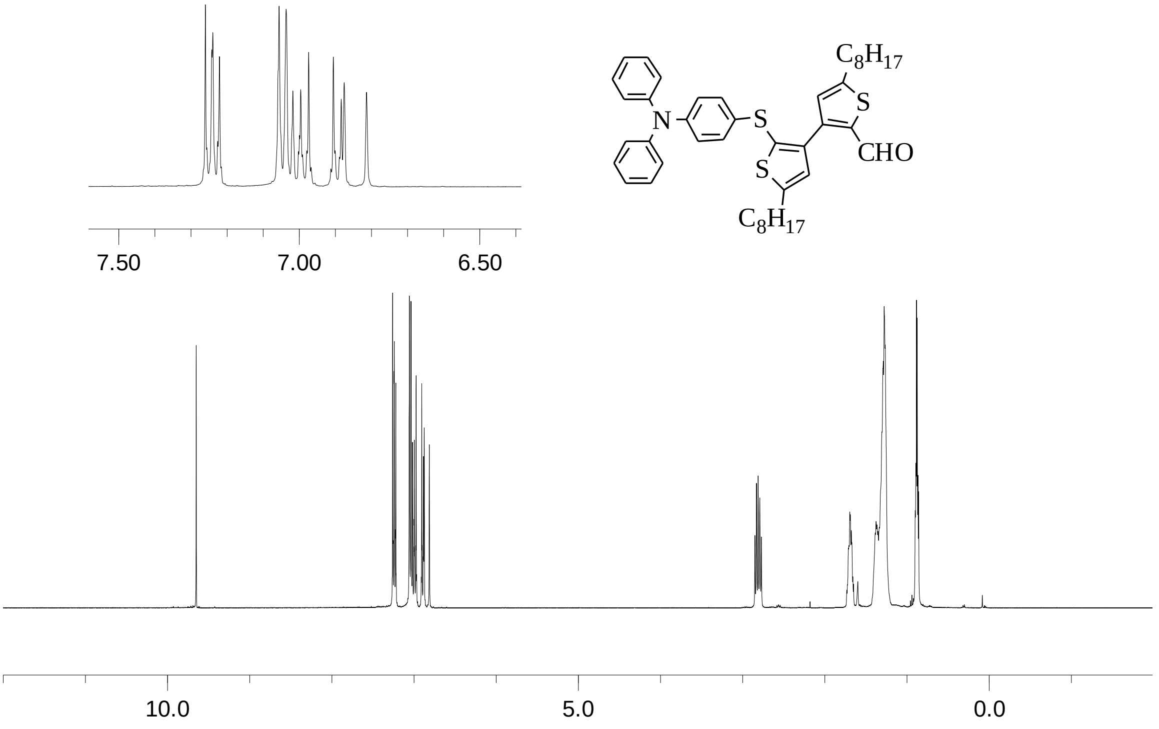

Figure S22: ^{1}H NMR (400 MHz, $CDCl_3$) spectrum of **3h**.

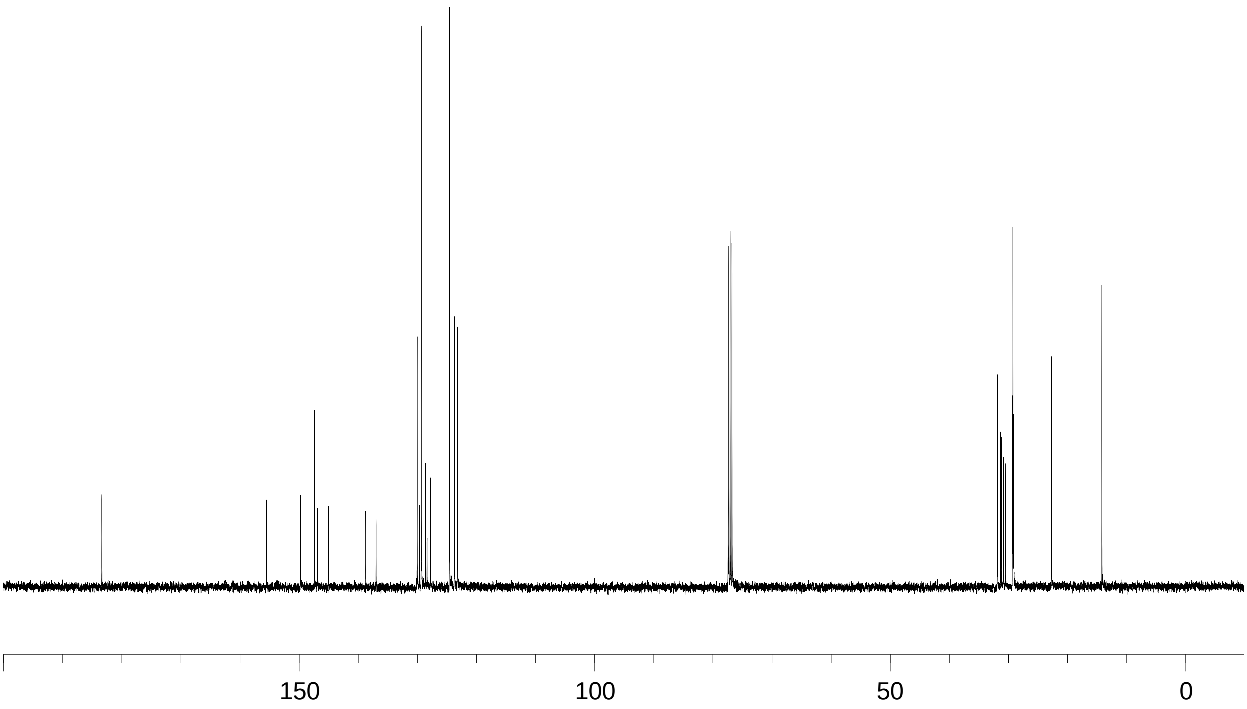

Figure S23: ^{13}C NMR (100 MHz, $CDCl_3$) spectrum of **3h**.

Instrument: IonSpec 4.7 Tesla FTMS

Card Serial Number : WI12 0427

Sample Serial Number: zh-2-61-col

Operator : HuaQin Date: 2012/03/22

Operation Mode: MALDI/DHB

Elemental Composition Search Report:

Target Mass:
Target m/z = 716.3037 ± 0.003
Charge = +1

Possible Elements:

Element:	Exact Mass:	Min:	Max:
C	12.000000	0	100
H	1.007825	0	100
N	14.003074	0	3
O	15.994915	0	3
S	31.972071	0	3
Na	22.989770	1	1

Additional Search Restrictions:
DBE Limit Mode = Both Integer and Half-Integer
Minimum DBE = 0

Search Results:
Number of Hits = 5

m/z	Delta m/z	DBE	Formula
716.30362	0.00008	34.0	$C_{51}H_{39}N_3Na^{+1}$
716.30431	-0.00061	24.5	$C_{45}H_{45}N_2O_3SNa^{+1}$
716.30250	0.00120	19.0	$C_{43}H_{51}NOS_3Na^{+1}$
716.30496	-0.00126	33.5	$C_{53}H_{41}ONa^{+1}$
716.30094	0.00276	29.5	$C_{48}H_{41}N_2O_3Na^{+1}$

Figure S24: HRMS data of **3h**.

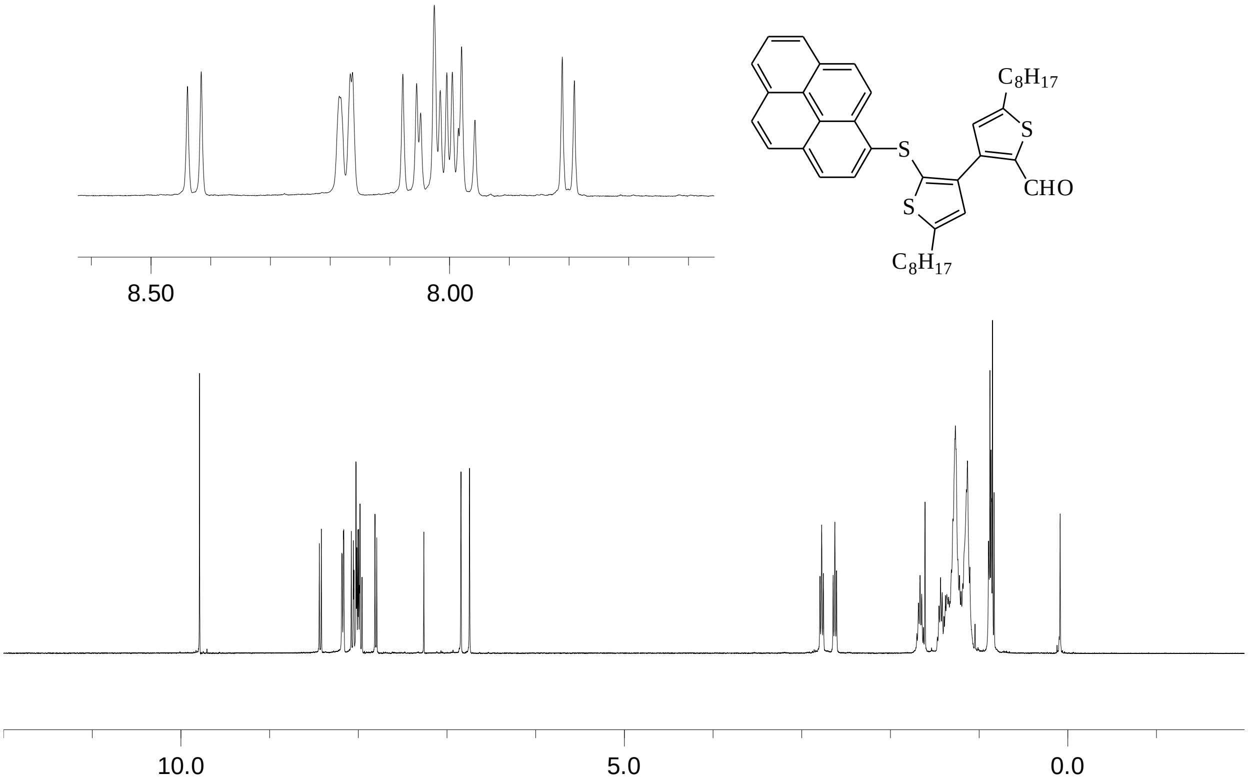

Figure S25: ^{1}H NMR (400 MHz, $CDCl_3$) spectrum of **3i**.

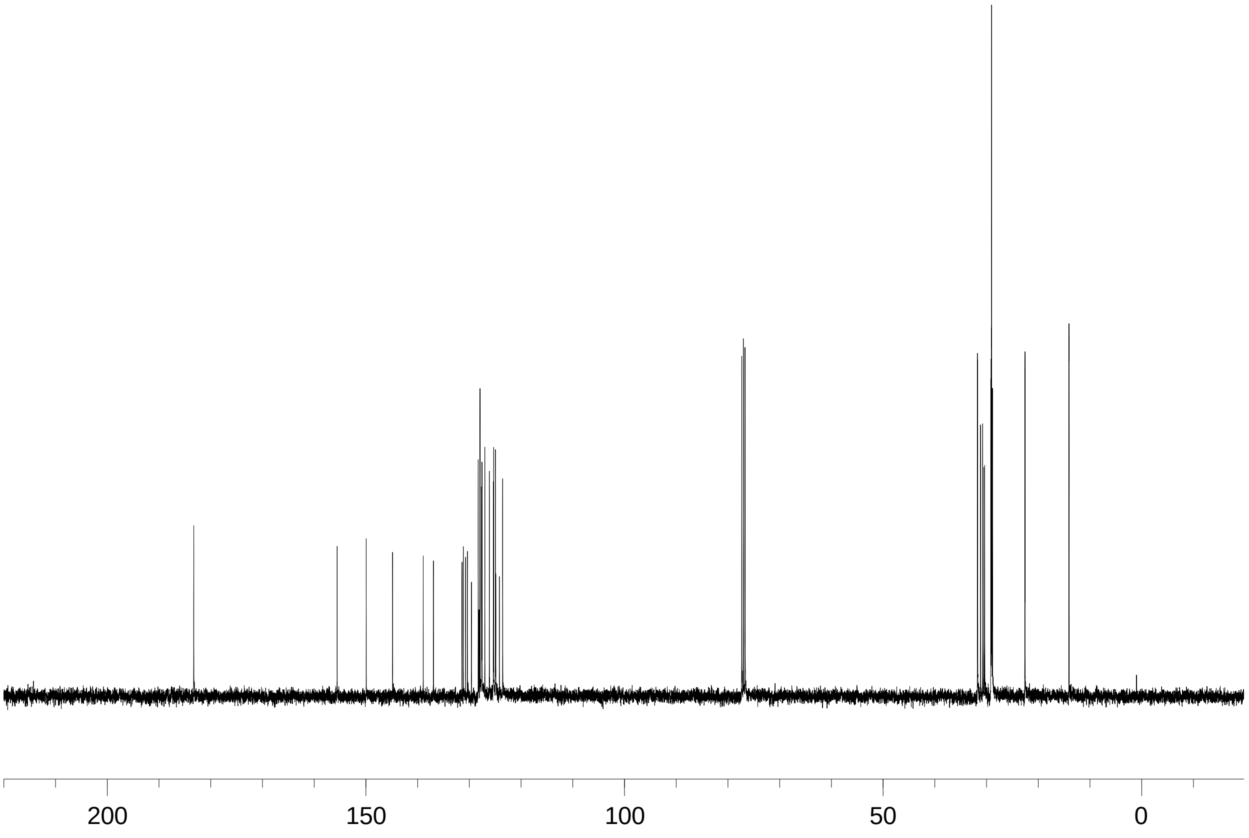

Figure S26: ^{13}C NMR (100 MHz, $CDCl_3$) spectrum of **3i**.

Instrument: IonSpec 4.7 Tesla FTMS

Card Serial Number : WI12 0655

Sample Serial Number: zh-3-27-col

Operator : HuaQin Date: 2012/05/15

Operation Mode: MALDI/DHB

Elemental Composition Search Report:

Target Mass:

Target m/z = 673.2611 ± 0.002
Charge = +1

Possible Elements:

Element:	Exact Mass:	Min:	Max:
C	12.000000	0	100
H	1.007825	0	100
O	15.994915	0	3
S	31.972071	0	3
Na	22.989770	0	1

Additional Search Restrictions:

DBE Limit Mode = Both Integer and Half-Integer
Minimum DBE = 0

Search Results:

Number of Hits = 3

m/z	Delta m/z	DBE	Formula
673.26030	0.00080	19.0	$C_{41}H_{46}OS_3Na^{+1}$
673.26270	-0.00160	21.5	$C_{43}H_{45}OS_3^{+1}$
673.25933	0.00177	26.5	$C_{46}H_{41}OS_2^{+1}$

Figure S27: HRMS data of **3i**.

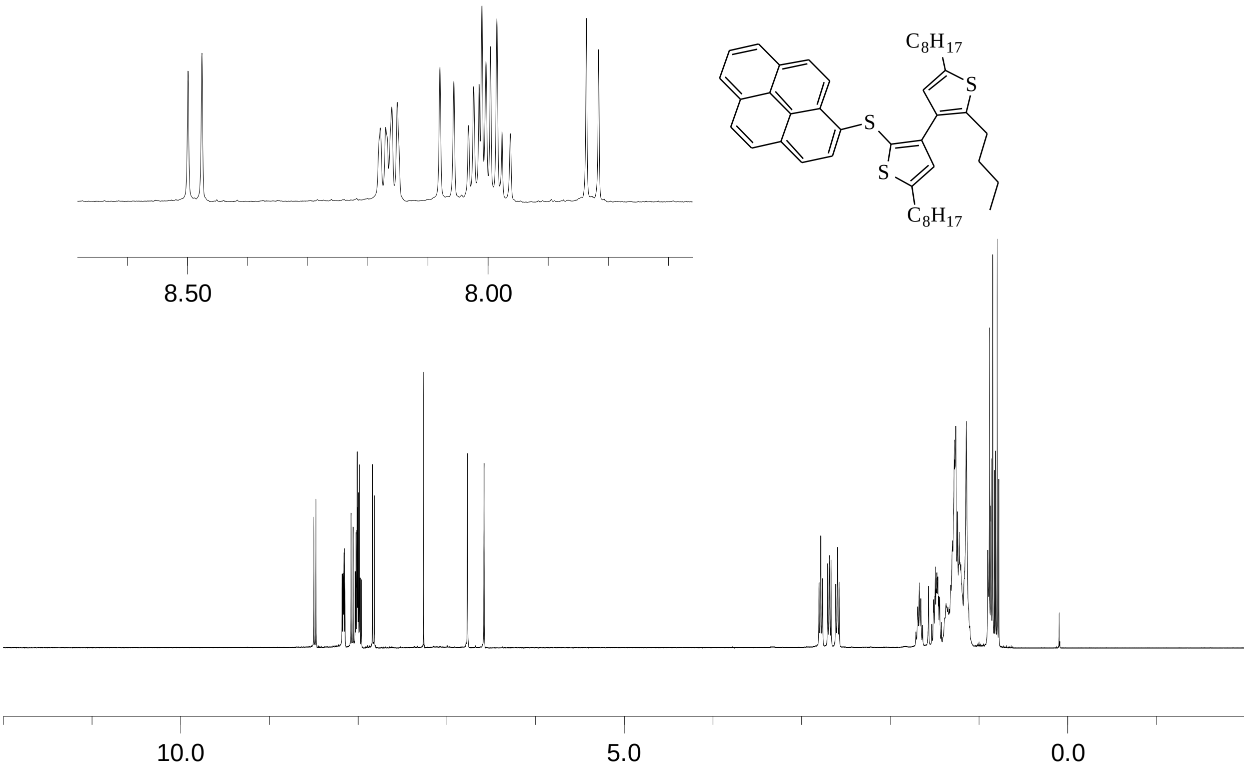

Figure S28: ^{1}H NMR (400 MHz, $CDCl_3$) spectrum of **4**.

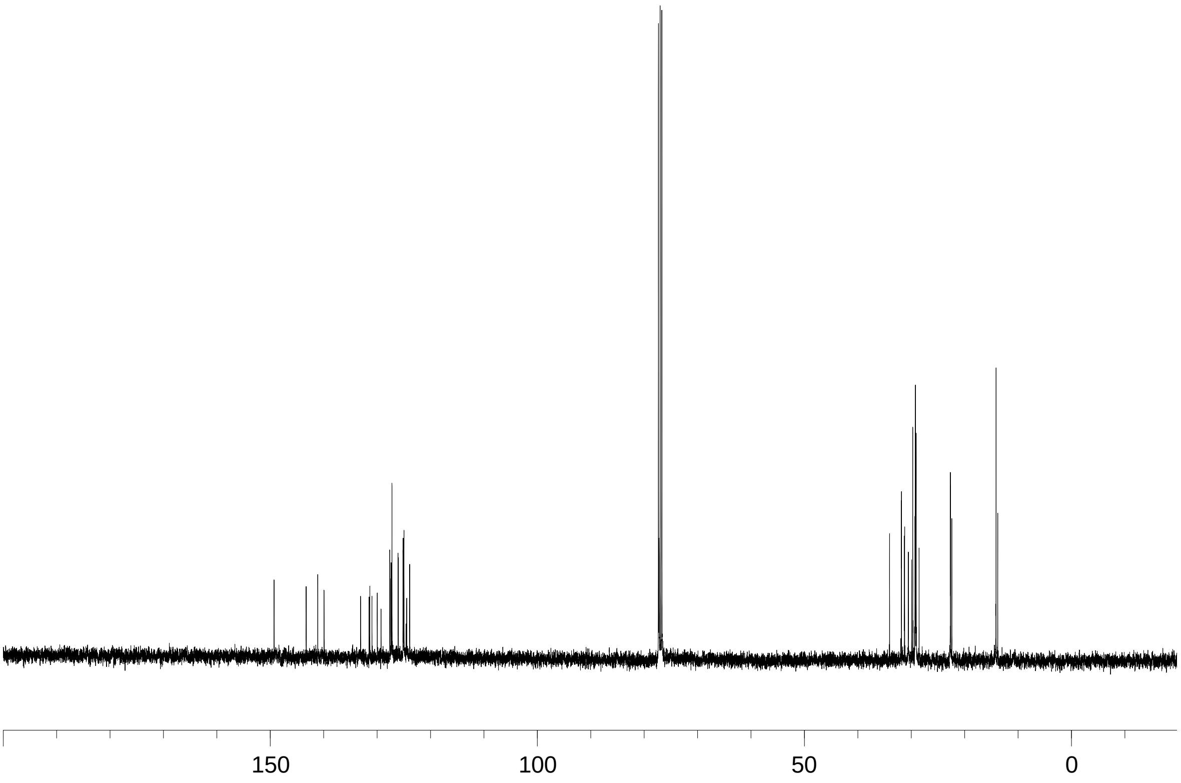

Figure S29: ^{13}C NMR (100 MHz, $CDCl_3$) spectrum of **4**.

Instrument: IonSpec 4.7 Tesla FTMS

Card Serial Number : WI12 0428

Sample Serial Number: zh-2-65-col2

Operator : HuaQin Date: 2012/03/22

Operation Mode: MALDI/DHB

Elemental Composition Search Report:

Target Mass:

Target m/z = 678.3392 ± 0.003

Charge = +1

Possible Elements:

Element:	Exact Mass:	Min:	Max:
C	12.000000	0	100
H	1.007825	0	100
S	31.972071	0	100

Additional Search Restrictions:

DBE Limit Mode = Both Integer and Half-Integer

Minimum DBE = 0

Search Results:

Number of Hits = 2

m/z	Delta m/z	DBE	Formula
678.33821	0.00099	18.0	$C_{44}H_{54}S_3^{+1}$
678.34159	-0.00239	13.0	$C_{41}H_{58}S_4^{+1}$

Figure S30: HRMS data of **4**.

5. X-ray crystallographic Data for 3i

Table S1: Crystal data and structure refinement for **3i**.

Identification code	**3i**
Empirical formula	$C_{41}H_{46}OS_3$
Formula weight	650.96
Temperature	296(2) K
Wavelength	0.71073 A
Crystal system, spacegroup	Triclinic,P-1
Unit cell dimensions	a = 9.3945(16) A alpha = 83.241(4) deg
	b = 11.3837(19) A beta = 79.382(4) deg
	c = 18.540(3) A gamma = 67.287(3) deg
Volume	1795.3(5) A^3
Z, Calculated density	2, 1.204 Mg/m^3
Absorption coefficient	0.237 mm^-1
F(000)	696
Crystal size	0.44 x 0.35 x 0.11 mm
Theta range for data collection	1.94 to 25.00deg.
Limiting indices	-11<=h<=9,-13<=k<=13,-20
Reflections collected/unique	9141/6253 [R(int)=0.0509]
Completeness to theta=25.00	98.90 %
Absorption correction	Semi-empirical from equivalent
Max.and min.transmission	0.9744 and 0.9028
Refinement method	Full-matrix least-squareson
Data/restraints/parameters	6253/30/380
Goodness-of-fit on F^2	1.063
Final R indices [I>2sigma(I)]	R1 = 0.0805, wR2 = 0.1802
R indices (all data)	R1 = 0.2076, wR2 = 0.2037
Largest diff.peak and hole	0.431 and -0.326 e.A^-3

Table S2: Atomic coordinates (x 10^4) and equivalent isotropic displacement parameters (A^2 x 10^3) for **3i.** U(eq) is defined as one third of the trace of the orthogonalized Uij tensor.

	x	y	z	U(eq)
O(1)	7401(7)	1800(5)	2307(3)	121(2)
S(1)	7763(2)	2131(2)	658(1)	88(1)
S(2)	13545(2)	1744(2)	473(1)	89(1)
S(3)	13161(3)	3535(2)	1625(1)	109(1)
C(1)	8688(8)	2512(6)	1257(4)	73(2)
C(2)	8776(8)	2647(6)	-89(4)	77(2)
C(3)	9771(8)	3085(6)	136(5)	83(2)
C(4)	9753(8)	3010(6)	885(4)	73(2)
C(5)	10785(10)	3389(7)	1241(4)	75(2)
C(6)	12394(10)	2902(6)	1073(4)	79(2)
C(7)	11379(12)	4458(7)	2064(4)	103(3)
C(8)	10266(10)	4267(7)	1790(4)	94(2)
C(9)	8362(10)	2244(7)	2027(5)	95(2)
C(10)	13688(8)	2645(6)	-364(4)	80(2)

C(11)	14040(8)	3731(7)	-388(5)	89(2)
C(12)	14200(9)	4409(7)	-1039(6)	95(3)
C(13)	14032(8)	4031(7)	-1680(5)	86(2)
C(14)	13659(8)	2953(6)	-1670(5)	77(2)
C(15)	13492(7)	2235(6)	-1006(4)	71(2)
C(16)	13094(8)	1141(6)	-1031(4)	86(2)
C(17)	12895(9)	797(7)	-1658(5)	93(2)
C(18)	13030(9)	1497(8)	-2319(5)	93(2)
C(19)	13448(9)	2572(7)	-2332(5)	89(2)
C(20)	13598(10)	3283(9)	-3001(6)	105(3)
C(21)	13355(12)	2886(11)	-3631(6)	134(4)
C(22)	12956(12)	1842(12)	-3613(6)	141(4)
C(23)	12803(11)	1122(9)	-2969(6)	126(3)
C(24)	14205(11)	4735(8)	-2357(6)	116(3)
C(25)	14004(11)	4382(9)	-2970(6)	120(3)
C(26)	8540(9)	2582(7)	-847(4)	94(2)
C(27)	8293(9)	1411(7)	-1010(4)	91(2)
C(28)	8158(9)	1417(7)	-1813(4)	100(2)
C(29)	7765(9)	358(6)	-2006(4)	96(2)
C(30)	7726(10)	362(7)	-2821(4)	112(3)
C(31)	7295(11)	-652(8)	-3042(5)	129(3)
C(32)	7191(15)	-554(11)	-3862(5)	188(5)
C(33)	6657(19)	-1512(13)	-4057(7)	272(8)
C(34)	11183(12)	5386(7)	2615(5)	159(4)
C(35)	11548(10)	4898(6)	3314(5)	225(7)
C(36)	11244(7)	6001(5)	3809(4)	251(8)
C(37)	12124(11)	5518(5)	4371(4)	333(11)
C(38)	11493(9)	6489(9)	4944(4)	343(8)
C(39)	12681(8)	6915(7)	5041(4)	349(7)
C(40)	12240(10)	7560(9)	5766(4)	383(8)
C(41)	12683(16)	8613(11)	5666(5)	372(9)

Table S3: Bond lengths [Å] and angles [˚] for **3i**.

O(1)-C(9)	1.208(8)	C(12)-C(13)-C(14)	119.5(8)
S(1)-C(2)	1.710(7)	C(12)-C(13)-C(24)	120.9(8)
S(1)-C(1)	1.715(6)	C(14)-C(13)-C(24)	119.6(9)
S(2)-C(6)	1.716(7)	C(13)-C(14)-C(15)	120.7(8)
S(2)-C(10)	1.770(7)	C(13)-C(14)-C(19)	119.8(8)
S(3)-C(7)	1.706(9)	C(15)-C(14)-C(19)	119.6(7)
S(3)-C(6)	1.711(7)	C(10)-C(15)-C(14)	118.1(6)
C(1)-C(4)	1.374(8)	C(10)-C(15)-C(16)	123.9(7)
C(1)-C(9)	1.426(9)	C(14)-C(15)-C(16)	118.0(7)
C(2)-C(3)	1.360(8)	C(17)-C(16)-C(15)	121.7(7)
C(2)-C(26)	1.476(8)	C(17)-C(16)-H(16A)	119.1
C(3)-C(4)	1.379(8)	C(15)-C(16)-H(16A)	119.1
C(3)-H(3A)	0.93	C(16)-C(17)-C(18)	121.6(7)
C(4)-C(5)	1.480(9)	C(16)-C(17)-H(17A)	119.2
C(5)-C(6)	1.381(9)	C(18)-C(17)-H(17A)	119.2

C(5)-C(8)	1.397(9)	C(17)-C(18)-C(23)	120.5(8)
C(7)-C(8)	1.340(9)	C(17)-C(18)-C(19)	119.3(8)
C(7)-C(34)	1.493(9)	C(23)-C(18)-C(19)	120.2(8)
C(8)-H(8A)	0.93	C(20)-C(19)-C(18)	119.7(9)
C(9)-H(9A)	0.93	C(20)-C(19)-C(14)	120.5(8)
C(10)-C(11)	1.392(8)	C(18)-C(19)-C(14)	119.8(8)
C(10)-C(15)	1.396(8)	C(21)-C(20)-C(19)	118.4(9)
C(11)-C(12)	1.370(9)	C(21)-C(20)-C(25)	125.1(10)
C(11)-H(11A)	0.93	C(19)-C(20)-C(25)	116.5(9)
C(12)-C(13)	1.366(9)	C(22)-C(21)-C(20)	121.6(9)
C(12)-H(12A)	0.93	C(22)-C(21)-H(21A)	119.2
C(13)-C(14)	1.398(9)	C(20)-C(21)-H(21A)	119.2
C(13)-C(24)	1.426(10)	C(21)-C(22)-C(23)	121.7(10)
C(14)-C(15)	1.414(8)	C(21)-C(22)-H(22A)	119.2
C(14)-C(19)	1.420(9)	C(23)-C(22)-H(22A)	119.2
C(15)-C(16)	1.439(8)	C(22)-C(23)-C(18)	118.5(9)
C(16)-C(17)	1.336(8)	C(22)-C(23)-H(23A)	120.8
C(16)-H(16A)	0.93	C(18)-C(23)-H(23A)	120.8
C(17)-C(18)	1.394(9)	C(25)-C(24)-C(13)	120.5(9)
C(17)-H(17A)	0.93	C(25)-C(24)-H(24A)	119.8
C(18)-C(23)	1.403(10)	C(13)-C(24)-H(24A)	119.8
C(18)-C(19)	1.419(9)	C(24)-C(25)-C(20)	123.1(9)
C(19)-C(20)	1.415(10)	C(24)-C(25)-H(25A)	118.4
C(20)-C(21)	1.385(11)	C(20)-C(25)-H(25A)	118.4
C(20)-C(25)	1.452(11)	C(2)-C(26)-C(27)	116.4(6)
C(21)-C(22)	1.373(11)	C(2)-C(26)-H(26A)	108.2
C(21)-H(21A)	0.93	C(27)-C(26)-H(26A)	108.2
C(22)-C(23)	1.384(11)	C(2)-C(26)-H(26B)	108.2
C(22)-H(22A)	0.93	C(27)-C(26)-H(26B)	108.2
C(23)-H(23A)	0.93	H(26A)-C(26)-H(26B)	107.4
C(24)-C(25)	1.316(10)	C(26)-C(27)-C(28)	111.5(6)
C(24)-H(24A)	0.93	C(26)-C(27)-H(27A)	109.3
C(25)-H(25A)	0.93	C(28)-C(27)-H(27A)	109.3
C(26)-C(27)	1.511(8)	C(26)-C(27)-H(27B)	109.3
C(26)-H(26A)	0.97	C(28)-C(27)-H(27B)	109.3
C(26)-H(26B)	0.97	H(27A)-C(27)-H(27B)	108
C(27)-C(28)	1.518(8)	C(29)-C(28)-C(27)	114.9(6)
C(27)-H(27A)	0.97	C(29)-C(28)-H(28A)	108.5
C(27)-H(27B)	0.97	C(27)-C(28)-H(28A)	108.5
C(28)-C(29)	1.487(8)	C(29)-C(28)-H(28B)	108.5
C(28)-H(28A)	0.97	C(27)-C(28)-H(28B)	108.5
C(28)-H(28B)	0.97	H(28A)-C(28)-H(28B)	107.5
C(29)-C(30)	1.517(8)	C(28)-C(29)-C(30)	113.0(6)
C(29)-H(29A)	0.97	C(28)-C(29)-H(29A)	109
C(29)-H(29B)	0.97	C(30)-C(29)-H(29A)	109
C(30)-C(31)	1.483(9)	C(28)-C(29)-H(29B)	109
C(30)-H(30A)	0.97	C(30)-C(29)-H(29B)	109
C(30)-H(30B)	0.97	H(29A)-C(29)-H(29B)	107.8

C(31)-C(32)	1.529(11)	C(31)-C(30)-C(29)	115.4(7)
C(31)-H(31A)	0.97	C(31)-C(30)-H(30A)	108.4
C(31)-H(31B)	0.97	C(29)-C(30)-H(30A)	108.4
C(32)-C(33)	1.468(13)	C(31)-C(30)-H(30B)	108.4
C(32)-H(32A)	0.97	C(29)-C(30)-H(30B)	108.4
C(32)-H(32B)	0.97	H(30A)-C(30)-H(30B)	107.5
C(33)-H(33A)	0.96	C(30)-C(31)-C(32)	112.9(8)
C(33)-H(33B)	0.96	C(30)-C(31)-H(31A)	109
C(33)-H(33C)	0.96	C(32)-C(31)-H(31A)	109
C(34)-C(35)	1.397(2)	C(30)-C(31)-H(31B)	109
C(34)-H(34A)	0.97	C(32)-C(31)-H(31B)	109
C(34)-H(34B)	0.97	H(31A)-C(31)-H(31B)	107.8
C(35)-C(36)	1.548(3)	C(33)-C(32)-C(31)	111.8(10)
C(35)-H(35A)	0.97	C(33)-C(32)-H(32A)	109.2
C(35)-H(35B)	0.97	C(31)-C(32)-H(32A)	109.2
C(36)-C(37)	1.379(2)	C(33)-C(32)-H(32B)	109.2
C(36)-H(36A)	0.97	C(31)-C(32)-H(32B)	109.2
C(36)-H(36B)	0.97	H(32A)-C(32)-H(32B)	107.9
C(37)-C(38)	1.498(2)	C(32)-C(33)-H(33A)	109.5
C(37)-H(37A)	0.97	C(32)-C(33)-H(33B)	109.5
C(37)-H(37B)	0.97	H(33A)-C(33)-H(33B)	109.5
C(38)-C(39)	1.423(2)	C(32)-C(33)-H(33C)	109.5
C(38)-H(38A)	0.97	H(33A)-C(33)-H(33C)	109.5
C(38)-H(38B)	0.97	H(33B)-C(33)-H(33C)	109.5
C(39)-C(40)	1.519(3)	C(35)-C(34)-C(7)	117.7(5)
C(39)-H(39A)	0.97	C(35)-C(34)-H(34A)	107.9
C(39)-H(39B)	0.97	C(7)-C(34)-H(34A)	107.9
C(40)-C(41)	1.395(2)	C(35)-C(34)-H(34B)	107.9
C(40)-H(40A)	0.97	C(7)-C(34)-H(34B)	107.9
C(40)-H(40B)	0.97	H(34A)-C(34)-H(34B)	107.2
C(41)-H(41A)	0.96	C(34)-C(35)-C(36)	110.1
C(41)-H(41B)	0.96	C(34)-C(35)-H(35A)	109.6
C(41)-H(41C)	0.96	C(36)-C(35)-H(35A)	109.6
C(2)-S(1)-C(1)	92.4(4)	C(34)-C(35)-H(35B)	109.6
C(6)-S(2)-C(10)	102.3(3)	C(36)-C(35)-H(35B)	109.6
C(7)-S(3)-C(6)	93.9(4)	H(35A)-C(35)-H(35B)	108.2
C(4)-C(1)-C(9)	128.8(7)	C(37)-C(36)-C(35)	108.3
C(4)-C(1)-S(1)	110.8(6)	C(37)-C(36)-H(36A)	110
C(9)-C(1)-S(1)	120.3(5)	C(35)-C(36)-H(36A)	110
C(3)-C(2)-C(26)	128.3(7)	C(37)-C(36)-H(36B)	110
C(3)-C(2)-S(1)	109.7(6)	C(35)-C(36)-H(36B)	110
C(26)-C(2)-S(1)	122.0(6)	H(36A)-C(36)-H(36B)	108.4
C(2)-C(3)-C(4)	115.3(7)	C(36)-C(37)-C(38)	107
C(2)-C(3)-H(3A)	122.3	C(36)-C(37)-H(37A)	110.3
C(4)-C(3)-H(3A)	122.3	C(38)-C(37)-H(37A)	110.3
C(1)-C(4)-C(3)	111.8(7)	C(36)-C(37)-H(37B)	110.3
C(1)-C(4)-C(5)	124.3(7)	C(38)-C(37)-H(37B)	110.3
C(3)-C(4)-C(5)	123.9(7)	H(37A)-C(37)-H(37B)	108.6

C(6)-C(5)-C(8)	111.6(7)	C(39)-C(38)-C(37)	109.6
C(6)-C(5)-C(4)	123.8(7)	C(39)-C(38)-H(38A)	109.8
C(8)-C(5)-C(4)	124.6(8)	C(37)-C(38)-H(38A)	109.8
C(5)-C(6)-S(3)	109.5(6)	C(39)-C(38)-H(38B)	109.8
C(5)-C(6)-S(2)	128.0(6)	C(37)-C(38)-H(38B)	109.8
S(3)-C(6)-S(2)	122.3(5)	H(38A)-C(38)-H(38B)	108.2
C(8)-C(7)-C(34)	128.2(9)	C(38)-C(39)-C(40)	109.7
C(8)-C(7)-S(3)	108.9(6)	C(38)-C(39)-H(39A)	109.7
C(34)-C(7)-S(3)	122.8(7)	C(40)-C(39)-H(39A)	109.7
C(7)-C(8)-C(5)	116.1(8)	C(38)-C(39)-H(39B)	109.7
C(7)-C(8)-H(8A)	122	C(40)-C(39)-H(39B)	109.7
C(5)-C(8)-H(8A)	122	H(39A)-C(39)-H(39B)	108.2
O(1)-C(9)-C(1)	124.1(8)	C(41)-C(40)-C(39)	108.6
O(1)-C(9)-H(9A)	117.9	C(41)-C(40)-H(40A)	110
C(1)-C(9)-H(9A)	117.9	C(39)-C(40)-H(40A)	110
C(11)-C(10)-C(15)	120.1(7)	C(41)-C(40)-H(40B)	110
C(11)-C(10)-S(2)	120.5(7)	C(39)-C(40)-H(40B)	110
C(15)-C(10)-S(2)	119.4(5)	H(40A)-C(40)-H(40B)	108.4
C(12)-C(11)-C(10)	120.8(7)	C(40)-C(41)-H(41A)	109.5
C(12)-C(11)-H(11A)	119.6	C(40)-C(41)-H(41B)	109.5
C(10)-C(11)-H(11A)	119.6	H(41A)-C(41)-H(41B)	109.5
C(13)-C(12)-C(11)	120.9(7)	C(40)-C(41)-H(41C)	109.5
C(13)-C(12)-H(12A)	119.6	H(41A)-C(41)-H(41C)	109.5
C(11)-C(12)-H(12A)	119.6	H(41B)-C(41)-H(41C)	109.5

Symmetry transformations used to generate equivalent atoms:

Table S4: Anisotropic displacement parameters (A^2 x 10^3) for **3i**.
The anisotropic displacement factor exponent takes the form:
-2 pi^2 [h^2 a*^2 U11 + ... + 2 h k a* b* U12]

	U11	U22	U33	U23	U13	U12
O(1)	161(6)	129(5)	102(4)	-9(3)	7(4)	-97(4)
S(1)	88(2)	90(1)	100(2)	-8(1)	-11(1)	-49(1)
S(2)	84(2)	80(1)	100(2)	0(1)	-10(1)	-29(1)
S(3)	119(2)	136(2)	106(2)	-7(1)	-27(2)	-79(2)
C(1)	71(5)	72(5)	83(6)	-12(4)	-8(5)	-35(4)
C(2)	69(5)	82(5)	83(5)	-6(4)	-15(4)	-31(4)
C(3)	73(6)	80(5)	101(7)	0(4)	-13(5)	-34(4)
C(4)	68(5)	65(4)	84(6)	-9(4)	-12(5)	-21(4)
C(5)	86(6)	72(5)	76(5)	-7(4)	-9(5)	-40(5)
C(6)	78(6)	80(5)	88(5)	-1(4)	-19(5)	-39(5)
C(7)	144(9)	106(6)	83(6)	-25(5)	-15(6)	-67(6)
C(8)	95(7)	92(6)	102(6)	-11(5)	-14(5)	-41(5)
C(9)	112(8)	85(6)	98(7)	-7(5)	-8(6)	-50(5)
C(10)	67(5)	65(5)	111(6)	0(4)	-7(5)	-31(4)
C(11)	76(6)	86(5)	117(7)	-7(5)	-15(5)	-43(5)

C(12)	78(6)	62(5)	144(8)	-4(6)	-2(6)	-33(4)
C(13)	67(5)	71(5)	115(7)	0(5)	8(5)	-31(4)
C(14)	66(5)	63(5)	92(6)	-7(5)	5(5)	-21(4)
C(15)	61(5)	63(5)	89(6)	-7(4)	-3(4)	-25(4)
C(16)	96(6)	60(5)	100(6)	0(4)	0(5)	-36(4)
C(17)	99(7)	82(6)	107(7)	-22(6)	-2(6)	-44(5)
C(18)	108(7)	84(6)	86(7)	-15(5)	-1(5)	-39(5)
C(19)	85(6)	80(6)	90(7)	-4(5)	5(5)	-28(5)
C(20)	101(7)	93(7)	96(8)	1(6)	4(6)	-21(5)
C(21)	160(10)	132(9)	88(8)	3(7)	1(7)	-43(8)
C(22)	170(11)	146(10)	109(9)	-31(8)	-13(7)	-57(8)
C(23)	160(10)	117(7)	101(7)	-28(7)	0(7)	-56(7)
C(24)	105(8)	89(7)	150(9)	12(7)	-7(7)	-43(6)
C(25)	115(8)	92(7)	131(9)	24(6)	10(7)	-36(6)
C(26)	82(6)	100(6)	105(7)	-5(5)	-25(5)	-34(5)
C(27)	95(6)	95(6)	87(6)	-17(4)	-12(5)	-38(5)
C(28)	106(7)	94(6)	105(7)	-11(5)	-15(5)	-41(5)
C(29)	104(7)	82(5)	95(6)	-12(4)	-16(5)	-24(5)
C(30)	140(8)	109(7)	85(6)	-14(5)	-22(6)	-38(6)
C(31)	133(9)	110(7)	138(9)	-23(6)	-34(7)	-30(6)
C(32)	242(15)	215(12)	124(9)	-40(8)	-72(9)	-75(11)
C(33)	360(20)	260(17)	230(14)	-77(12)	-93(14)	-111(15)
C(34)	221(13)	158(9)	133(8)	-16(8)	-26(9)	-108(9)
C(35)	330(20)	190(12)	161(11)	-36(10)	-61(12)	-83(12)
C(36)	283(19)	252(15)	192(13)	-120(12)	-64(12)	-29(13)
C(37)	330(30)	370(20)	280(20)	-132(16)	-95(16)	-60(19)
C(38)	349(13)	364(12)	312(11)	-119(9)	-40(10)	-104(10)
C(40)	360(11)	337(11)	306(11)	96(10)	53(10)	-48(10)

Table S5: Hydrogen coordinates (x 10^4) and isotropic displacement parameters (A^2 x 10^3) for **3i**.

	x	y	z	U(eq)
H(3A)	10418	3413	-193	100
H(8A)	9210	4693	1955	113
H(9A)	8925	2423	2334	114
H(11A)	14168	3999	42	107
H(12A)	14426	5137	-1043	114
H(16A)	12973	661	-599	103
H(17A)	12661	73	-1653	112
H(21A)	13463	3338	-4075	161
H(22A)	12785	1612	-4045	170
H(23A)	12555	405	-2967	151
H(24A)	14461	5449	-2366	139
H(25A)	14127	4860	-3403	144
H(26A)	9442	2635	-1180	113
H(26B)	7642	3327	-955	113

H(27A)	7348	1378	-707	109
H(27B)	9161	654	-885	109
H(28A)	7362	2219	-1945	120
H(28B)	9141	1377	-2108	120
H(29A)	6753	422	-1735	116
H(29B)	8530	-448	-1855	116
H(30A)	6988	1183	-2971	135
H(30B)	8748	279	-3087	135
H(31A)	6294	-601	-2762	154
H(31B)	8065	-1478	-2922	154
H(32A)	6473	291	-3990	225
H(32B)	8210	-669	-4143	225
H(33A)	6586	-1411	-4573	408
H(33B)	5649	-1402	-3779	408
H(33C)	7386	-2350	-3948	408
H(34A)	11824	5875	2412	191
H(34B)	10104	5976	2673	191
H(35A)	12636	4332	3275	270
H(35B)	10914	4411	3533	270
H(36A)	11536	6666	3523	301
H(36B)	10145	6365	4009	301
H(37A)	13213	5368	4190	400
H(37B)	12043	4718	4575	400
H(38A)	10610	7206	4794	412
H(38B)	11138	6116	5405	412
H(39A)	12808	7512	4639	418
H(39B)	13666	6196	5039	418
H(40A)	11123	7832	5925	460
H(40B)	12767	6967	6141	460
H(41A)	12407	9032	6120	558
H(41B)	12155	9196	5296	558
H(41C)	13791	8335	5514	558
